# The vicious cycle of frailty and pain: a two-sided causal relationship revealed

**DOI:** 10.3389/fmed.2024.1396328

**Published:** 2024-09-09

**Authors:** Ruipeng Zhong, Yijian Chen, Lanhua Zhong, Guiming Huang, Weidong Liang, Yun Zou

**Affiliations:** ^1^Department of Anesthesiology, Ganzhou People’s Hospital, Ganzhou, China; ^2^Anesthesia Surgery Center, the First Affiliated Hospital of Gannan Medical University, Ganzhou, China

**Keywords:** aging, frailty, pain, causal effect, Mendelian randomization

## Abstract

**Background:**

The decline in physiological functions in the older people is frequently accompanied with pain and frailty, yet the causal connection between frailty and pain remains uncertain. In this study, we utilized a two-sample Mendelian randomization (MR) approach to investigate the potential causal association between frailty and pain.

**Methods:**

Two-sample bidirectional MR was conducted using summary data from genome-wide association studies to examine the potential causal relationship between frailty (defined by the frailty index and frailty phenotype) and pain. Summary genome wide association statistics were extracted from populations of European ancestry. We also investigated the causal relationship between frailty and site-specific pain, including joint pain, limb pain, thoracic spine pain and low back pain. Causal effects were estimated using the inverse variance weighting method. Sensitivity analyses were performed to validate the robustness of the results.

**Results:**

Genetic predisposition to frailty was associated with an increased risk of pain (frailty phenotype odds ratio [OR]: 1.73; *P* = 3.54 × 10^–6^, frailty index OR: 1.36; *P* = 2.43 × 10^–4^). Meanwhile, individuals with a genetic inclination toward pain had a higher risk of developing frailty. Regarding site-specific pain, genetic prediction of the frailty phenotype increased the occurrence risk of joint pain, limb pain and low back pain. Reverse MR analysis further showed that limb pain and low back pain were associated with an increased risk of frailty occurrence.

**Conclusion:**

This study presented evidence supporting a bidirectional causal relationship between frailty and pain. We highlighted the significance of addressing pain to prevent frailty and recommend the inclusion of pain assessment in the evaluation system for frailty.

## Introduction

With the rapid global aging phenomenon, geriatric medicine is encountering unprecedented challenges. Frailty, as a common clinical syndrome in the older people, draws significant attention in the field of public health (1). The primary characteristic of frailty is the decline in physiological system functions, accompanied with an increase in vulnerability to stressors. Frailty has been confirmed to be associated with various adverse events, such as falls, disabilities, mental disorders and mortality (2). Preoperative frailty in patients can further lead to poor outcomes, increased consumption of healthcare resources, and elevated medical costs (3). Pain is the most common cause of impairment in daily activities and is one of the most prevalent and burdensome conditions affecting the overall quality of life (4).

Pain and frailty are associated with the decline in physiological functions, exerting negative impacts on the quality of life of the older people (5). Furthermore, the prevalence of pain and frailty tends to increase with age (6, 7). Observational studies suggest a bidirectional relationship between pain and frailty, indicating a potential vicious cycle where each condition accelerates the development of the other (8). However, observational studies are susceptible to confounding factors and reverse causation, creating a challenge in understanding the relationship between frailty and pain. This dilemma raises the question of whether to treat pain to prevent or reverse frailty or to manage frailty to prevent or reduce pain. Clarifying the causal relationship between frailty and pain is crucial to developing highly targeted interventions, thereby promoting population health.

**FIGURE 1:**
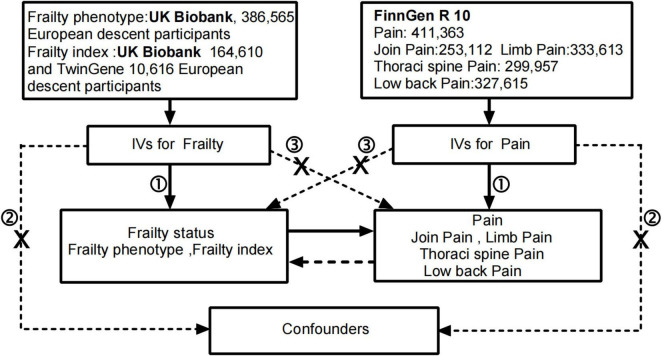
The flowchart in this Mendelian randomization study. Conducting MR studies requires meeting three key assumptions: the genetic variants ① are associated with the exposure, ② are independent of confounders and ③ affect the outcome through the exposure of interestonly. IVs, instrumental variables.

Mendelian randomization (MR) uses genetic variations that are strongly correlated with the exposure factor as instrumental variables to assess the causal relationship between the exposure factor and the outcome. Since genetic variants are randomly assigned to the entire population at meiosis and conception, it satisfies both the rationality of temporal order and is less susceptible to traditional confounding factors such as environment and behavior (9). Compared with traditional observational studies, this approach maximally avoids or reduces the impact of confounding factors and prevents reverse causation (10). In the present study, we utilized summary statistics from an independent genome-wide association study (GWAS) for two-sample MR analysis to investigate the causal relationship between frailty and pain. Conducting MR studies requires meeting three key assumptions. Firstly, the chosen instrumental variables should exhibit a significant correlation with the exposure. Secondly, instrumental variables should be unrelated to potential confounding factors between exposure and outcome. Thirdly, instrumental variables should have no direct relationship with the outcome and be causally linked only through exposure (11). Figure 1 illustrates the flowchart of our study process.

## Materials and methods

### Study design

This study conducted a two-sample MR analysis based on the Strengthening the Reporting of Observational studies in Epidemiology–MR guidelines (12). The summary statistics used in the study are publicly available, and all original studies obtained ethical approval and informed consent from the respective institutions, obviating the need for additional ethical applications.

### Frailty and pain GWAS data sources

Frailty phenotype and frailty index are the most extensively used frailty-assessment tools that have been validated by multiple large-scale cohort studies (1). The frailty index and frailty phenotype have different conceptual bases, but they share commonalities in determining factors and identifying frailty (13). The assessment content of the frailty phenotype includes five criteria, namely, weight loss, exhaustion, low physical activity, slow walking speed, and weak grip strength, and it is defined as frailty when the performance reaches three criteria. The GWAS summary data of the frailty phenotype originate from the most recent large-scale GAWS study involving 386,565 participants of European ancestry, with an average age of 57 years and 54% women (14). The summary data of the frailty index come from the latest GWAS meta-analysis, which included 166,410 participants of European ancestry from the UK Biobank (aged between 60 and 70 years) and 10,616 participants from Sweden (aged between 41 and 87 years). The calculation of the frailty index was based on 49 and 44 self-reported symptoms, disabilities and diagnosed diseases from the UK Biobank and TwinGene, respectively. This study estimated the Single Nucleotide Polymorphism (SNP) heritability of frailty to be 11% (15).

To reduce the bias caused by sample overlap, we selected the pain GWAS data from the latest Finnish database R10 version, The FinnGen study, which is a large-scale genomics initiative that has analyzed over 500,000 Finnish biobank samples, correlating genetic variation with health data to understand disease mechanisms and predispositions (16). The current work also extracted pain data from different parts of the body, including joint pain, limb pain, low back pain and thoracic spine pain. The GWAS data were corrected for age, gender and the first 10 genetic principal components. Detailed information is obtained in the Supplementary Table.

### Statistical analysis

#### Selection of instrumental variables

We conducted a bidirectional two-sample Mendelian randomization study to investigate the effect of frailty on pain first (forward MR) and the effect of pain on frailty in the second step (reverse MR). Forward MR used frailty as the exposure factor and pain as the outcome. Using *P* < 5 × 10^–8^ as the screening criterion, SNPs with statistical significance from the GWAS summary data of the study were selected as the preliminary screened instrumental variables. The linkage disequilibrium coefficient r^2^ was set to <0.001 and the region width was 10,000 kb to ensure that the SNPs were independent of one another and to exclude the influence of gene pleiotropy on the results (17). The online tool^1^ was utilized to eliminate SNPs that were significantly associated with confounding factors. We also excluded SNPs that were closely related to the outcome (*P* < 5 × 10^–5^). The F statistic was used to quantify the strength of the instrumental variables. We did not seek out proxy SNPs for missing SNPs. Finally, the exposure and outcome data were coordinated, and palindromic SNPs were deleted. When the resulting instrumental variables were less than 3, we set the exposure related to *P* < 5 × 10^–6^ to include additional SNPs for MR analysis. Reverse MR used pain as the exposure factor and frailty as the outcome, and the screening process was the same as that for forward MR.

#### Two-sample MR analysis

Inverse-variance weighted method (IVW) was the main method used in this MR study. IVW weights each variant by taking the inverse of the variance of the effect estimate for each genetic variant as a weight, resulting in a comprehensive effect estimate, it can provide robust causal assessment in the absence of directional pleiotropy (18). To avoid the bias of IVW results caused by horizontal pleiotropy of any SNP, we used MR–Egger and weighted median to enhance the robustness of the results. When the instrumental variables had pleiotropy, the IVW estimates may be biased, so we conducted sensitivity analysis to further confirm the reliability of the results. Cochran Q test heterogeneity (19), if the Cochran Q statistic test has statistical significance, indicating that the analysis results have significant heterogeneity, then focus on the results of the random effects IVW method. MR-PRESSO is a method for MR analysis that assesses the effect of each SNP on the outcome variable by modeling the distribution to detect any violation of the assumption of independence. We used the MR-PRESSO method to further search for sources of heterogeneity, and re-analyses were conducted after excluding the instrumental variables with obvious heterogeneity. The Egger bias intercept test was used to quantitatively detect horizontal pleiotropy. Leave-one sensitivity analysis was performed for single SNP analysis to determine whether the association between genetic variation and fragility is affected by single SNPs.

For binary outcomes, odds ratios (ORs) and 95% CIs were used to estimate the degree of causal relationship. All *p*-values were two-tailed. The above methods were implemented in the ‘gwasglue’ and ‘TwoSampleMR’ packages in R 4.2.1 software.

## Results

### Frailty effect on pain

**FIGURE 2:**
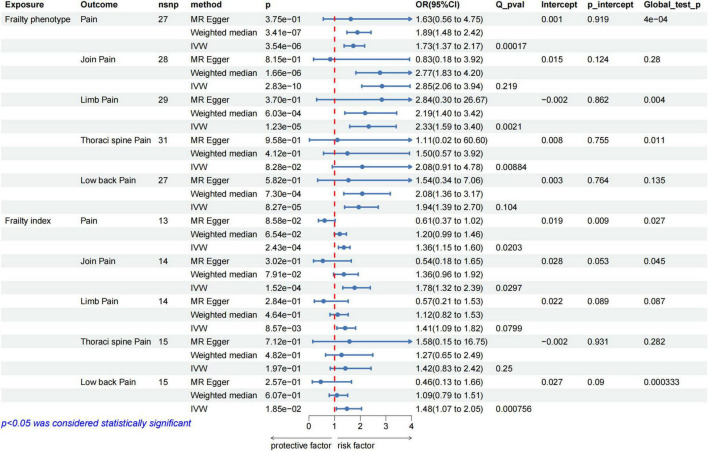
MR analysis evaluated the effects of frailty on pain. IVW, inverse variance weighted; OR, odds ratio; CI, confidence interval; Q_pval, Cochran Q test *p*-value; p_intercept, MR-Egger regression *p*-value.

**FIGURE 3:**
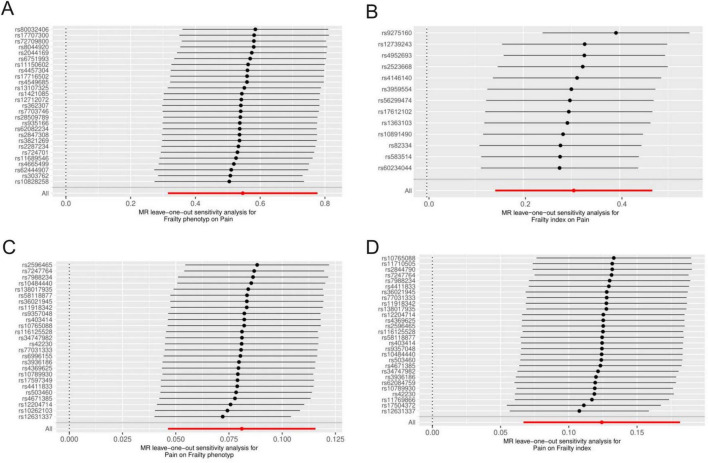
Results of the leave-one-out in MR analysis were used to judge the stability of the results. Each line above represents the result of MR analysis after deleting the SNP. The bottom line represents the results of the overall MR analysis. **(A)** Frailty phenotype on Pain; **(B)** frailty index on pain; **(C)** pain on frailty phenotype; **(D)** pain on frailty index.

After rigorous screening, 27 and 13 independent SNPs were selected as instrumental variables for the frailty phenotype and frailty index, respectively, in a two-sample MR analysis. All instrumental variables (IVs) exhibited F statistics greater than 10, indicating a low risk of weak instrument bias. Detailed information on the SNPs is provided in Supplementary Tables 1, 2. IVW results showed that genetically predicted frailty was associated with increased risk of pain (frailty phenotype OR: 1.73, 95% CI: 1.37–2.17, *P* = 3.54 × 10^–6^; frailty index OR: 1.36, 95% CI: 1.15–1.6, *P* = 2.43 × 10^–4^). The weighted-median method yielded similar results (Figure 2). Cochran’s Q heterogeneity test showed significant heterogeneity amongst the instrumental variables, and the MR–PRESSO method remained significant after removing the outliers with evident heterogeneity. MR–Egger analysis did not detect directional pleiotropy amongst the instrumental variables (Figure 2). Finally, the study performed sensitivity analysis using the leave-one-out method. All lines were on one side of the y-axis even after removing single SNPs, which verified the stability of our results (Figures 3A, B).

The results of further analysis of the causal relationship between the genetically predicted frailty phenotype and frailty index with pain in different body parts are depicted in Figure 2. Frailty was associated with an increased risk of joint pain, lower back pain and limb pain, whereas no correlation was observed with thoracic spine pain. Cochran’s Q heterogeneity test indicated heterogeneity amongst instrumental variables, and MR–Egger analysis did not detect directional pleiotropy within the instrumental variables (Figure 2). Sensitivity analysis using the leave-one-out method did not reveal substantial influence from individual SNPs on the results. The Supplementary Figure shows the corresponding results.

### Pain effect on frailty

**FIGURE 4:**
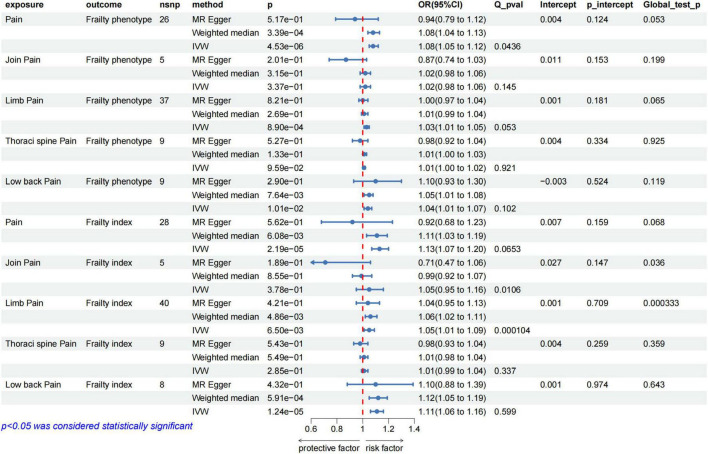
MR analysis evaluated the effects of pain on frailty. IVW, inverse variance weighted; OR, odds ratio; CI, confidence interval; Q_pval, Cochran Q test *p*-value; p_intercept, MR-Egger regression *p*-value.

We selected 5–40 independent SNPs as instrumental variables for pain, thoracic pain, limb pain, back pain, and joint pain. All IVs had *F*-values greater than 10, indicating low risk of weak instrument bias. The details of the SNPs are provided in the Supplementary Tables 3–7. IVW results showed that genetically predicted pain, limb pain and low back pain were associated with the increased risk of frailty, whereas thoracic pain and joint pain were not. The weighted median method showed similar results (Figure 4). Cochran’s Q heterogeneity test showed no significant heterogeneity amongst the IVs, except for pain and limb pain. After removing the IVs with significant heterogeneity using the MR–PRESSO method, the *p*-values remained significant. MR–Egger analysis did not detect any directional pleiotropy in the IVs, and the causal relationships were robust (Figure 4). Leave-one-out sensitivity analysis did not find any single SNP to have a significant influence on the results (Figures 3C, D). The Supplementary Figure shows the results for specific pain sites.

## Discussion

This study is the first to elucidate the relationship between frailty and pain using MR methods. Genetically predicted frailty and pain were found to have a bidirectional causal relationship. For pain in specific body parts, we found that genetically predicted frailty increased the risk of joint pain, limb pain and low back pain. We also revealed that limb pain and back pain were associated with increased risk of frailty.

Pain is an important risk factor for frailty, and a systematic review of 23 studies has shown that older people with any pain have a significantly higher likelihood of developing frailty than those who reported no pain (OR: 3.38; 95% CI: 2.65 and 4.31) (20). Recent population studies from Europe, Asia and America have also demonstrated that pain promotes frailty in older people (21–23). Notably, the frequency and intensity of pain significantly affect the onset and progression of frailty (24). According to Wade et al., in their 8-year study involving 5,316 older people individuals, after controlling for variables such as age, gender, BMI, lifestyle and depression, seniors with moderate or severe pain were found to have a higher risk of frailty (OR values of 3.08 and 3.78, respectively) compared with their counterparts. The severity of pain in older people is also dose-dependently associated with the occurrence of frailty (25). Furthermore, observations in hospitalized older patients have revealed a correlation between chronic pain, somatic pain and widespread pain and severe frailty (26). The present study supported the causal relationship between pain and frailty. For pain in specific body parts, we found that limb pain and back pain had a causal relationship with frailty, similar to the conclusion of Rocha et al. (27). However, our findings were contrary to that of Chaplin et al., who argued that groups with joint pain are more likely to become frail than those without (8). This conclusion is easy to understand because exercise and nutrition are the main interventions to prevent or reverse frailty (28); limb pain, thoracic pain, back pain or joint pain can limit the exercise of these groups to varying degrees, especially limb pain and back pain. Our results did not statistically support a causal relationship amongst thoracic pain, joint pain, and frailty statistically, which may be related to the insufficient statistical power of the samples.

Pain may be a consequence of frailty (29). A cohort study from Investigating Musculoskeletal Health and Wellbeing has shown that after adjusting for baseline pain, gender, age and body mass index, baseline frailty remains associated with the severity of joint pain 1 year later, and frailty may have a small to moderate effect on future joint pain (8). The trajectory of knee joint pain over a 9-year period in patients with frailty has been studied by CAI, confirming the significant role of frailty in the development of knee joint pain (30). Older patients with cancer often experience frailty and chronic pain and frailty status is associated with persistent pain and pain intensity in hospitalized patients with cancer (31). Some studies have also shown that a strong link remains between preoperative frailty and postoperative chronic pain even after adjusting for comorbidities, preoperative pain and type of surgery (32). We confirmed the causal relationship between frailty and pain based on GWAS data, including the causal relationship between frailty and joint pain, limb pain, and back pain. However, a longitudinal study from Australia has revealed that chronic pain increases the risk of frailty, but the frailty status is unrelated to future chronic or invasive pain (33). This contradictory result may be due to the inclusion of only men in the study, whereas women are generally considered to be an independent risk factor for chronic pain (34). Frail individuals are also associated with limited life expectancy, and frail individuals are highly likely to be lost during follow-up (35).

The bidirectional causal relationship between frailty and pain may be influenced by multiple physiological mechanisms, such as pain homeostasis imbalance (36, 37), hypothalamic–pituitary–adrenal axis dysfunction (38) and immune-inflammatory response (39), which are related to the decline in the body’s ability to resist internal and external environmental stress. The main characteristic of frailty is the weakening of stress resistance. This effect also partly explains the dose–response relationship between pain intensity and frailty, that is, a more severe pain corresponds with more activity and autonomy of older people decline, leading to frailty. Some studies have further suggested that chronic pain and frailty may have common genetic characteristics and neural pathways. Researchers have proposed that weight, depression, sleep and other factors mediate the relationship between chronic pain and frailty (40). Few studies have focused on the effect of frailty on pain and the specific mechanism requires require further investigation. The possible mechanisms are that patients with frailty become more cautious in using analgesics, leading to more prominent pain problems, compared with their counterparts (41). Impaired activity, depression, reduced nutritional intake and other potential mechanisms may also cause chronic pain (37).

Frailty and pain are not permanent states, and clinical practice can improve frailty and pain through effective interventions (42). Our study confirmed the bidirectional causal relationship between frailty and pain, which may have important implications for clinical practice. We support incorporating pain relief strategies into interventions aimed at preventing, delaying or managing frailty, as well as including pain in the measurement and assessment system of frailty (8, 43). This technique can help us better identify and manage frail populations and ultimately improve the care of patients with pain and frailty and those at increased risk.

The strength of this study was in the utilization of an MR design, which effectively minimized the impact of confounding factors and prevented reverse causation. The frailty phenotype and frailty index GWAS data were used for analysis, making the results convincing. The study population was all European, reducing heterogeneity. The GWAS data of exposure and outcome originated from different databases, reducing the bias caused by sample overlap. However, our study had many limitations. Firstly, the selected data did not stratify the nature of pain, and different types of pain may have varying effects on frailty status (44). Secondly, no definite standard exists for the assessment of frailty, and the methods of frailty evaluation used in different studies are inconsistent, which may cause bias in our interpretation of results. Thirdly, the bidirectional causal relationship between frailty and pain may be influenced by various intermediate mediators, and our study did not explore the biological mechanism of the relationship between frailty and pain. Finally, the study comprised European populations, and the differences in results amongst various populations need to be verified.

## Conclusion

In summary, this study provided evidence of a bidirectional causal relationship between frailty and pain. We emphasized that frailty should be prevented by resolving pain, and pain should be incorporated into the assessment system of frailty. An important perspective was provided for reducing the burden of coexisting pain and frailty, which may help optimize the care of older adults.

## Data Availability

The original contributions presented in this study are included in this article/Supplementary material, further inquiries can be directed to the corresponding author.

## Footnotes

1
www.phenoscanner.medschl.cam.ac.uk
