# Supplementary material for: The vicious cycle of frailty and pain: a two-sided causal relationship revealed
Source: Front Med (Lausanne). 2024 Sep 9;11:1396328. doi: 10.3389/fmed.2024.1396328 (PMC11416971; doi:10.3389/fmed.2024.1396328)
Supplement: Supplementary file 1 [file Table_1.DOCX]

Supplementary Tables for

**The Vicious Cycle of Frailty and Pain: A Two-Sided Causal Relationship Revealed**

Yun Zou, Yijian Chen, Lanhua Zhong, Guiming Huang, Chuanwu Zhang, Weidong Liang, Ruipeng Zhong

Table S: Summary of the Genome-Wide Association Study Datasets

Table S1: Genetic instruments for Frailty phenotype

Table S2: Genetic instruments for Frailty index

Table S3: Genetic instruments for Pain

Table S4: Genetic instruments for Join Pain

Table S5: Genetic instruments for Limb Pain

Table S6: Genetic instruments for Thoracic spine Pain

Table S7: Genetic instruments for Low back pain

Table S8: Genetic predisposition to Frailty and risk of Pain: the results from Mendelian randomization analysis

Table S9: Genetic predisposition to Pain and risk of Frailty: the results from Mendelian randomization analysis

| **Table S :Summary of the Genome-Wide Association Study Datasets** | | | | | |
| --- | --- | --- | --- | --- | --- |
| Phenotype | Study | Data sources | Ancestry | Sample size(case/control) | DOI/URL |
| Frailty phenotype | YE Y et al,2023 | UK Biobank | European | 386,565(NA/NA) | [10.1007/s11357-023-00771-z](https://doi.org/10.1007/s11357-023-00771-z) |
| Frailty index | ATKINS J et al,2021 | UK Biobank and TwinGene | European | 175 226(NA/NA) | [10.1111/acel.13459](https://doi.org/10.1111/acel.13459) |
| Pain | Kurki et al,2023 | FinnGen R10 | European | 411,363(189683/221680) | https://storage.googleapis.com/finngen-public-data-r10/summary_stats/finngen_R10_PAIN.gz |
| Join Pain | Kurki et al,2023 | FinnGen R10 | European | (30614/222498) | https://storage.googleapis.com/finngen-public-data-r10/summary_stats/finngen_R10_JOINTPAIN.gz |
| Limb Pain | Kurki et al,2023 | FinnGen R10 | European | (34007/299606) | https://storage.googleapis.com/finngen-public-data-r10/summary_stats/finngen_R10_M13_LIMBPAIN.gz |
| Thoracic spine Pain | Kurki et al,2023 | FinnGen R10 | European | (5187/294770) | https://storage.googleapis.com/finngen-public-data-r10/summary_stats/finngen_R10_M13_THORACISPINEPAIN.gz |
| Low back pain | Kurki et al,2023 | FinnGen R10 | European | (32845/294770) | https://storage.googleapis.com/finngen-public-data-r10/summary_stats/finngen_R10_M13_LOWBACKPAIN.gz |

| **TableS1 .Genetc instruments for Frailty phenotype** | | | | | | | | | |
| --- | --- | --- | --- | --- | --- | --- | --- | --- | --- |
|  | SNP | effect_allele | other_allele | beta | se | pval | N | R2 | F |
| 1 | rs6751993 | A | G | -0.0145464 | 0.00245869 | 2.50E-09 | 386565 | 9.05402E-05 | 35.00285764 |
| 2 | rs4665499 | G | A | -0.0108039 | 0.00183069 | 4.30E-09 | 386565 | 9.00886E-05 | 34.82823715 |
| 3 | rs11689546 | A | G | -0.0116917 | 0.001862 | 2.90E-10 | 386565 | 0.000101983 | 39.42720337 |
| 4 | rs935166 | G | A | 0.0110031 | 0.00183021 | 2.20E-09 | 386565 | 9.349E-05 | 36.14333908 |
| 5 | rs2287234 | C | A | 0.0105934 | 0.00184425 | 1.10E-08 | 386565 | 8.53437E-05 | 32.99371041 |
| 6 | rs79659409 | G | C | 0.0167463 | 0.00288146 | 5.70E-09 | 386565 | 8.73679E-05 | 33.77633659 |
| 7 | rs3821269 | A | G | 0.0109514 | 0.0018329 | 2.50E-09 | 386565 | 9.2342E-05 | 35.69946776 |
| 8 | rs12712072 | A | G | 0.0145931 | 0.0021564 | 1.60E-11 | 386565 | 0.000118457 | 45.79694104 |
| 9 | rs12992177 | C | G | -0.0164544 | 0.00299264 | 4.00E-08 | 386565 | 7.81985E-05 | 30.23118342 |
| 10 | rs11130207 | T | G | 0.0153427 | 0.00248901 | 5.30E-10 | 386565 | 9.82845E-05 | 37.99708703 |
| 11 | rs362307 | C | T | -0.0287299 | 0.00349173 | 1.50E-16 | 386565 | 0.000175101 | 67.69972743 |
| 12 | rs13107325 | C | T | -0.0309861 | 0.00349612 | 7.60E-19 | 386565 | 0.000203166 | 78.55271039 |
| 13 | rs7703746 | G | A | 0.0131507 | 0.00183162 | 6.40E-13 | 386565 | 0.000133336 | 51.54980027 |
| 14 | rs10053447 | T | C | 0.0123915 | 0.00187284 | 3.10E-11 | 386565 | 0.000113233 | 43.77704165 |
| 15 | rs2857597 | T | A | -0.0119924 | 0.0020349 | 3.40E-09 | 386565 | 8.98389E-05 | 34.73170215 |
| 16 | rs9273148 | T | A | -0.012091 | 0.00208969 | 7.70E-09 | 386565 | 8.65965E-05 | 33.47809315 |
| 17 | rs62444907 | C | T | 0.018619 | 0.00254562 | 3.10E-13 | 386565 | 0.00013837 | 53.49652288 |
| 18 | rs4549685 | C | T | 0.0109354 | 0.00194589 | 1.80E-08 | 386565 | 8.16911E-05 | 31.58150153 |
| 19 | rs4457304 | A | C | 0.0103236 | 0.00183334 | 2.00E-08 | 386565 | 8.20197E-05 | 31.70854469 |
| 20 | rs17716502 | C | T | 0.013064 | 0.00227635 | 9.00E-09 | 386565 | 8.51951E-05 | 32.93625891 |
| 21 | rs72709800 | G | A | -0.0208164 | 0.0033968 | 8.80E-10 | 386565 | 9.71419E-05 | 37.55530518 |
| 22 | rs10986468 | A | T | 0.0114702 | 0.0018388 | 4.10E-10 | 386565 | 0.000100648 | 38.91105365 |
| 23 | rs10828258 | A | G | -0.0141621 | 0.00196632 | 7.50E-13 | 386565 | 0.000134173 | 51.87366346 |
| 24 | rs2847308 | C | T | 0.0121126 | 0.00194858 | 5.90E-10 | 386565 | 9.99476E-05 | 38.64010249 |
| 25 | rs80032406 | T | C | -0.012647 | 0.00209359 | 1.30E-09 | 386565 | 9.43905E-05 | 36.49150429 |
| 26 | rs724701 | A | G | 0.0121211 | 0.00213607 | 1.00E-08 | 386565 | 8.32903E-05 | 32.19979503 |
| 27 | rs28509789 | T | G | 0.0118716 | 0.0021483 | 3.30E-08 | 386565 | 7.89899E-05 | 30.53716062 |
| 28 | rs17707300 | T | C | -0.011961 | 0.00188296 | 2.50E-10 | 386565 | 0.000104372 | 40.3508597 |
| 29 | rs11150602 | A | G | 0.0107475 | 0.00190908 | 2.00E-08 | 386565 | 8.19801E-05 | 31.69324091 |
| 30 | rs1421085 | T | C | -0.0129537 | 0.00186546 | 3.00E-12 | 386565 | 0.000124721 | 48.21873219 |
| 31 | rs8044920 | C | T | -0.0106959 | 0.00189191 | 1.60E-08 | 386565 | 8.26752E-05 | 31.96198345 |
| 32 | rs2044169 | A | G | -0.0111414 | 0.00202308 | 3.90E-08 | 386565 | 7.84507E-05 | 30.32867378 |
| 33 | rs12601919 | A | G | -0.0128805 | 0.00234357 | 3.20E-08 | 386565 | 7.81363E-05 | 30.2071383 |
| 34 | rs303762 | T | C | -0.0106475 | 0.00192983 | 3.80E-08 | 386565 | 7.87409E-05 | 30.44087913 |
| 35 | rs62082234 | T | G | -0.0105411 | 0.00191644 | 4.10E-08 | 386565 | 7.82573E-05 | 30.2539032 |
| 36 | rs9953231 | G | A | -0.0121973 | 0.0021889 | 3.00E-08 | 386565 | 8.0319E-05 | 31.0509984 |
| 37 | rs11660938 | G | T | -0.0105044 | 0.00186205 | 1.50E-08 | 386565 | 8.23193E-05 | 31.82437073 |
| 38 | rs660010 | G | C | 0.0173554 | 0.00260378 | 2.30E-11 | 386565 | 0.000114918 | 44.42840084 |

Note: SNP, single nucleotide polymorphism; eaf, effect allele frequency; R^2^ = (beta^2^) / (se^2^*N + beta^2^); F = beta^2^ / se^2^

| **Table S2. Genetic instruments for Frailty index** | | | | | | | | | | |
| --- | --- | --- | --- | --- | --- | --- | --- | --- | --- | --- |
| 1 | SNP | effect_allele | other_allele | eaf | beta | se | pval | N | R^2^ | F |
| 2 | rs12739243 | T | C | 0.2206 | 0.0242 | 0.004 | 1.28E-09 | 175226 | 0.000208844 | 36.6025 |
| 3 | rs4952693 | T | C | 0.3734 | -0.0194 | 0.0034 | 1.47E-08 | 175226 | 0.000185766 | 32.55709343 |
| 4 | rs583514 | T | C | 0.5111 | -0.0199 | 0.0033 | 1.65E-09 | 175226 | 0.000207486 | 36.36455464 |
| 5 | rs2071207 | T | C | 0.478 | 0.0187 | 0.0033 | 1.47E-08 | 175226 | 0.000183222 | 32.11111111 |
| 6 | rs82334 | A | C | 0.3177 | 0.0223 | 0.0035 | 3.13E-10 | 175226 | 0.000231619 | 40.59510204 |
| 7 | rs1363103 | T | C | 0.38 | 0.0191 | 0.0034 | 2.23E-08 | 175226 | 0.000180066 | 31.55795848 |
| 8 | rs2523668 | A | C | 0.1643 | 0.0326 | 0.0045 | 2.92E-13 | 175226 | 0.000299421 | 52.48197531 |
| 9 | rs555911977 | T | C | 0.2793 | 0.034 | 0.0046 | 2.10E-13 | 175226 | 0.00031168 | 54.63137996 |
| 10 | rs374943348 | A | T | 0.3605 | 0.0274 | 0.0039 | 3.23E-12 | 175226 | 0.000281612 | 49.35963182 |
| 11 | rs9275160 | A | G | 0.3397 | 0.0382 | 0.0035 | 7.18E-28 | 175226 | 0.000679355 | 119.1216327 |
| 12 | rs60234044 | T | C | 0.4733 | 0.0201 | 0.0033 | 1.22E-09 | 175226 | 0.000211677 | 37.09917355 |
| 13 | rs56299474 | A | C | 0.1733 | 0.0241 | 0.0044 | 3.94E-08 | 175226 | 0.000171181 | 30.00051653 |
| 14 | rs4146140 | T | C | 0.3811 | -0.0198 | 0.0034 | 6.83E-09 | 175226 | 0.000193504 | 33.91349481 |
| 15 | rs10891490 | T | C | 0.5915 | 0.0188 | 0.0034 | 2.00E-08 | 175226 | 0.000174455 | 30.57439446 |
| 16 | rs3959554 | A | G | 0.4177 | -0.0189 | 0.0034 | 1.74E-08 | 175226 | 0.000176316 | 30.90051903 |
| 17 | rs17612102 | T | C | 0.5933 | -0.0187 | 0.0034 | 2.85E-08 | 175226 | 0.000172604 | 30.25 |
| 18 | rs8089807 | T | C | 0.1866 | -0.0248 | 0.0043 | 6.50E-09 | 175226 | 0.000189795 | 33.26338561 |

Note: SNP, single nucleotide polymorphism; eaf, effect allele frequency; R^2^ = (beta^2^) / (se^2^*N + beta^2^); F = beta^2^ / se^2^

| **Table S3. Genetic instruments for Pain** | | | | | | | | | | |
| --- | --- | --- | --- | --- | --- | --- | --- | --- | --- | --- |
|  | SNP | other_allele | effect_allele | eaf | beta | se | pval | N | R^2^ | F |
| 1 | rs503460 | A | C | 0.649336 | 0.026658 | 0.00473336 | 1.78E-08 | 342161 | 9.26926E-05 | 31.71874931 |
| 2 | rs77031333 | C | T | 0.21531 | 0.0311121 | 0.00552034 | 1.74E-08 | 342161 | 9.28231E-05 | 31.76340106 |
| 3 | rs4671385 | T | C | 0.467205 | 0.0258634 | 0.00455422 | 1.35E-08 | 342161 | 9.42479E-05 | 32.25100264 |
| 4 | rs34747982 | G | A | 0.408833 | -0.0252678 | 0.0045973 | 3.88E-08 | 342161 | 8.82796E-05 | 30.20850075 |
| 5 | rs116125528 | C | T | 0.0724149 | -0.0527568 | 0.00875295 | 1.67E-09 | 342161 | 0.000106163 | 36.32854425 |
| 6 | rs12631337 | A | G | 0.606775 | -0.0275887 | 0.0046251 | 2.45E-09 | 342161 | 0.000103979 | 35.58116954 |
| 7 | rs11918342 | A | G | 0.314397 | -0.026676 | 0.00488055 | 4.61E-08 | 342161 | 8.73043E-05 | 29.87472283 |
| 8 | rs11710505 | G | A | 0.198483 | -0.0397796 | 0.00578836 | 6.32E-12 | 342161 | 0.000138013 | 47.2291094 |
| 9 | rs4411833 | A | G | 0.609339 | 0.0283083 | 0.00465208 | 1.16E-09 | 342161 | 0.000108207 | 37.02825363 |
| 10 | rs2178487 | T | A | 0.550652 | 0.0278103 | 0.00454927 | 9.77E-10 | 342161 | 0.000109207 | 37.37041506 |
| 11 | rs36021945 | T | C | 0.0997305 | -0.0442884 | 0.00757288 | 4.97E-09 | 342161 | 9.99503E-05 | 34.20249843 |
| 12 | rs10484440 | A | G | 0.187367 | -0.0360476 | 0.00581023 | 5.50E-10 | 342161 | 0.000112483 | 38.49160866 |
| 13 | rs9357048 | A | G | 0.14057 | 0.0422245 | 0.00649281 | 7.86E-11 | 342161 | 0.000123589 | 42.29252773 |
| 14 | rs111508444 | A | G | 0.19957 | -0.0479961 | 0.00576565 | 8.47E-17 | 342161 | 0.000202487 | 69.29714598 |
| 15 | rs2844790 | C | G | 0.135367 | 0.0410861 | 0.00660012 | 4.81E-10 | 342161 | 0.000113242 | 38.75129088 |
| 16 | rs2596465 | T | C | 0.492211 | 0.0284774 | 0.00452027 | 2.98E-10 | 342161 | 0.000115982 | 39.68916098 |
| 17 | rs3128759 | C | T | 0.644539 | -0.0306479 | 0.00472585 | 8.86E-11 | 342161 | 0.000122902 | 42.05731838 |
| 18 | rs403414 | A | G | 0.251091 | -0.0315799 | 0.00520834 | 1.33E-09 | 342161 | 0.000107435 | 36.76400754 |
| 19 | rs12204714 | C | T | 0.630906 | -0.0271767 | 0.00468312 | 6.51E-09 | 342161 | 9.84124E-05 | 33.67618356 |
| 20 | rs11769866 | G | A | 0.23608 | -0.0299319 | 0.00530772 | 1.71E-08 | 342161 | 9.29355E-05 | 31.80186302 |
| 21 | rs58118877 | C | T | 0.451557 | 0.0293077 | 0.00454714 | 1.15E-10 | 342161 | 0.000121396 | 41.5419447 |
| 22 | rs3936186 | G | A | 0.604034 | -0.0253468 | 0.00462722 | 4.31E-08 | 342161 | 8.76874E-05 | 30.00585313 |
| 23 | rs10262103 | C | A | 0.53899 | 0.0285626 | 0.00453761 | 3.08E-10 | 342161 | 0.000115787 | 39.62243333 |
| 24 | rs42230 | C | T | 0.54573 | -0.0251007 | 0.00454497 | 3.34E-08 | 342161 | 8.91334E-05 | 30.5006872 |
| 25 | rs138017935 | C | T | 0.0188145 | -0.094299 | 0.0166818 | 1.58E-08 | 342161 | 9.33807E-05 | 31.95422982 |
| 26 | rs6996155 | G | T | 0.479397 | -0.0271128 | 0.00453217 | 2.20E-09 | 342161 | 0.000104583 | 35.78791173 |
| 27 | rs112535944 | C | A | 0.210885 | 0.0326677 | 0.00557221 | 4.56E-09 | 342161 | 0.00010044 | 34.37020849 |
| 28 | rs10765088 | G | A | 0.356763 | -0.0267933 | 0.00471264 | 1.30E-08 | 342161 | 9.4461E-05 | 32.32390959 |
| 29 | rs10789930 | T | G | 0.263531 | 0.0298054 | 0.00514616 | 6.96E-09 | 342161 | 9.8028E-05 | 33.54465577 |
| 30 | rs7987501 | G | C | 0.456679 | -0.0249813 | 0.00453917 | 3.72E-08 | 342161 | 8.85132E-05 | 30.28845867 |
| 31 | rs7988234 | T | C | 0.350195 | -0.031764 | 0.00473255 | 1.92E-11 | 342161 | 0.000131641 | 45.04846037 |
| 32 | rs4369625 | G | A | 0.312361 | -0.0277518 | 0.00487758 | 1.27E-08 | 342161 | 9.46023E-05 | 32.37229665 |
| 33 | rs62084759 | A | G | 0.708459 | -0.0332969 | 0.00499096 | 2.53E-11 | 342161 | 0.000130063 | 44.50813792 |
| 34 | rs17597349 | C | T | 0.102627 | -0.0429649 | 0.00746198 | 8.52E-09 | 342161 | 9.68828E-05 | 33.15274165 |
| 35 | rs17504372 | C | T | 0.417394 | 0.0327167 | 0.00458549 | 9.69E-13 | 342161 | 0.000148755 | 50.90582522 |
| 36 | rs7247764 | T | C | 0.267681 | 0.0280998 | 0.00511369 | 3.91E-08 | 342161 | 8.82407E-05 | 30.19518285 |

Note: SNP, single nucleotide polymorphism; eaf, effect allele frequency; R^2^ = (beta^2^) / (se^2^*N + beta^2^); F = beta^2^ / se^2^

| **Table S4. Genetic instruments for Join Pain** | | | | | | | | | | |
| --- | --- | --- | --- | --- | --- | --- | --- | --- | --- | --- |
|  | SNP | other_allele | effect_allele | eaf | beta | se | pval | N | R^2^ | F |
| 1 | rs4655301 | T | C | 0.219214 | -0.0594022 | 0.0107988 | 3.78E-08 | 215181 | 0.000140601 | 30.25896462 |
| 2 | rs72965147 | A | G | 0.384288 | 0.0503894 | 0.00900507 | 2.20E-08 | 215181 | 0.000145491 | 31.31152269 |
| 3 | rs138585026 | G | A | 0.137141 | 0.0859803 | 0.0125281 | 6.74E-12 | 215181 | 0.000218841 | 47.10071389 |
| 4 | rs2844791 | G | A | 0.155033 | 0.0771021 | 0.0119925 | 1.28E-10 | 215181 | 0.000192055 | 41.33452579 |
| 5 | rs9267091 | G | A | 0.209422 | 0.0774445 | 0.0106905 | 4.35E-13 | 215181 | 0.000243823 | 52.4789485 |
| 6 | rs62444907 | C | T | 0.207756 | -0.0661911 | 0.0109275 | 1.38E-09 | 215181 | 0.000170482 | 36.69083257 |

Note: SNP, single nucleotide polymorphism; eaf, effect allele frequency; R^2^ = (beta^2^) / (se^2^*N + beta^2^); F = beta^2^ / se^2^

| **Table S5. Genetic instruments for Limb Pain** | | | | | | | | | | |
| --- | --- | --- | --- | --- | --- | --- | --- | --- | --- | --- |
|  | SNP | other_allele | effect_allele | eaf | beta | se | pval | N | R^2^ | F |
| 1 | rs79544310 | G | C | 0.0951347 | -0.069683 | 0.0144264 | 1.36E-06 | 278804 | 8.36763E-05 | 23.33123564 |
| 2 | rs1573066 | T | A | 1.54E-05 | 4.0251 | 0.874788 | 4.20E-06 | 278804 | 7.59304E-05 | 21.17130921 |
| 3 | rs71662956 | A | C | 0.111028 | 0.0606514 | 0.0128742 | 2.46E-06 | 278804 | 7.9599E-05 | 22.19428491 |
| 4 | rs1800601 | G | A | 0.642262 | -0.0435209 | 0.00852943 | 3.35E-07 | 278804 | 9.33719E-05 | 26.0348882 |
| 5 | rs489286 | A | G | 0.695147 | 0.0413858 | 0.00894582 | 3.72E-06 | 278804 | 7.67591E-05 | 21.40239615 |
| 6 | rs6025 | C | T | 0.0201631 | 0.241543 | 0.0274243 | 1.28E-18 | 278804 | 0.000278162 | 77.57429007 |
| 7 | rs4634891 | C | T | 0.514975 | -0.0397166 | 0.00820895 | 1.31E-06 | 278804 | 8.39525E-05 | 23.40824774 |
| 8 | rs76909456 | A | G | 0.265391 | -0.0459888 | 0.00931906 | 8.02E-07 | 278804 | 8.7342E-05 | 24.35341891 |
| 9 | rs1596747 | A | G | 0.498663 | 0.0397521 | 0.00818647 | 1.20E-06 | 278804 | 8.45651E-05 | 23.57907714 |
| 10 | rs74182380 | G | A | 0.0801185 | -0.0702437 | 0.0153336 | 4.63E-06 | 278804 | 7.52653E-05 | 20.98584685 |
| 11 | rs1850169 | A | T | 0.575115 | -0.0397874 | 0.0082507 | 1.42E-06 | 278804 | 8.34016E-05 | 23.25463502 |
| 12 | rs602928 | A | G | 0.19094 | 0.0493349 | 0.0103241 | 1.76E-06 | 278804 | 8.18973E-05 | 22.83516213 |
| 13 | rs3772858 | A | G | 0.0117329 | 0.168419 | 0.036495 | 3.93E-06 | 278804 | 7.63806E-05 | 21.29685298 |
| 14 | rs12641989 | G | A | 0.074405 | 0.0716302 | 0.0152616 | 2.69E-06 | 278804 | 7.90058E-05 | 22.02886869 |
| 15 | rs160045 | G | A | 0.262207 | -0.0448997 | 0.00937182 | 1.66E-06 | 278804 | 8.23198E-05 | 22.95297592 |
| 16 | rs72788523 | A | G | 0.25699 | 0.0436243 | 0.00932073 | 2.86E-06 | 278804 | 7.85641E-05 | 21.90569756 |
| 17 | rs34644295 | C | T | 0.408491 | 0.0382043 | 0.00832371 | 4.44E-06 | 278804 | 7.55542E-05 | 21.06641385 |
| 18 | rs72791979 | G | A | 0.044208 | -0.0949207 | 0.0205471 | 3.84E-06 | 278804 | 7.654E-05 | 21.34129629 |
| 19 | rs140751470 | A | C | 0.0181133 | 0.13551 | 0.029414 | 4.08E-06 | 278804 | 7.61206E-05 | 21.22435565 |
| 20 | rs6903679 | T | G | 0.870164 | 0.056348 | 0.0123228 | 4.82E-06 | 278804 | 7.49906E-05 | 20.90923829 |
| 21 | rs73728583 | G | A | 0.244487 | -0.0453709 | 0.00957137 | 2.13E-06 | 278804 | 8.05884E-05 | 22.47018139 |
| 22 | rs2246753 | G | A | 0.0969033 | 0.0648033 | 0.0137458 | 2.42E-06 | 278804 | 7.97114E-05 | 22.22563628 |
| 23 | rs145067054 | C | T | 0.357755 | 0.0398308 | 0.0085402 | 3.10E-06 | 278804 | 7.80134E-05 | 21.7521393 |
| 24 | rs78363798 | G | C | 0.109069 | -0.0608739 | 0.013305 | 4.76E-06 | 278804 | 7.5076E-05 | 20.93304925 |
| 25 | rs1058335 | C | T | 0.257923 | -0.0440761 | 0.00943971 | 3.02E-06 | 278804 | 7.81909E-05 | 21.8016325 |
| 26 | rs189972707 | C | T | 0.0575965 | 0.0804322 | 0.0173239 | 3.44E-06 | 278804 | 7.73101E-05 | 21.55601908 |
| 27 | rs8176645 | A | T | 0.541267 | -0.0483792 | 0.0081912 | 3.50E-09 | 278804 | 0.000125103 | 34.88368091 |
| 28 | rs138351956 | G | T | 0.0294737 | -0.115505 | 0.0249883 | 3.79E-06 | 278804 | 7.66295E-05 | 21.36624216 |
| 29 | rs72821151 | C | T | 0.0224355 | 0.137273 | 0.0267301 | 2.81E-07 | 278804 | 9.45865E-05 | 26.37357983 |
| 30 | rs10835074 | T | G | 0.608617 | -0.0398378 | 0.0084031 | 2.13E-06 | 278804 | 8.06079E-05 | 22.47561739 |
| 31 | rs523586 | C | T | 0.409355 | -0.0410296 | 0.00834742 | 8.87E-07 | 278804 | 8.6647E-05 | 24.15961643 |
| 32 | rs149948945 | G | T | 0.0863263 | 0.0673692 | 0.0145006 | 3.38E-06 | 278804 | 7.74138E-05 | 21.58493947 |
| 33 | rs638465 | G | C | 0.858934 | 0.0549612 | 0.0118734 | 3.68E-06 | 278804 | 7.68475E-05 | 21.42704171 |
| 34 | rs17114972 | G | A | 0.278174 | 0.0441964 | 0.00910541 | 1.21E-06 | 278804 | 8.44966E-05 | 23.55997248 |
| 35 | rs12579691 | G | A | 0.0238728 | -0.129621 | 0.0279453 | 3.51E-06 | 278804 | 7.71615E-05 | 21.51459538 |
| 36 | rs9597861 | A | G | 0.313473 | -0.0411447 | 0.00884543 | 3.29E-06 | 278804 | 7.75992E-05 | 21.63664404 |
| 37 | rs72686755 | G | T | 0.0528472 | -0.0855267 | 0.0186671 | 4.61E-06 | 278804 | 7.52867E-05 | 20.99180206 |
| 38 | rs12590613 | A | G | 0.153324 | -0.0541599 | 0.011461 | 2.29E-06 | 278804 | 8.00897E-05 | 22.33112911 |
| 39 | rs11158795 | C | T | 0.337137 | 0.0454453 | 0.00863528 | 1.42E-07 | 278804 | 9.93306E-05 | 27.6965134 |
| 40 | rs8033644 | A | G | 0.745971 | 0.0491201 | 0.00943174 | 1.91E-07 | 278804 | 9.72733E-05 | 27.12282014 |
| 41 | rs141322066 | T | C | 0.0447463 | -0.0983371 | 0.0203655 | 1.37E-06 | 278804 | 8.36198E-05 | 23.31549494 |
| 42 | rs115546269 | A | G | 0.00423625 | -0.332004 | 0.0707406 | 2.69E-06 | 278804 | 7.8998E-05 | 22.02668567 |
| 43 | rs77121361 | C | T | 0.0461689 | 0.0896352 | 0.0190997 | 2.69E-06 | 278804 | 7.89898E-05 | 22.02440031 |
| 44 | rs8045875 | T | C | 0.594358 | 0.042321 | 0.00834068 | 3.89E-07 | 278804 | 9.23357E-05 | 25.74595012 |
| 45 | rs59640213 | G | A | 0.28211 | 0.0519401 | 0.00907151 | 1.03E-08 | 278804 | 0.00011757 | 32.78282652 |
| 46 | rs72903983 | C | G | 0.0915862 | 0.0663638 | 0.014086 | 2.46E-06 | 278804 | 7.96074E-05 | 22.19663413 |
| 47 | rs234621 | C | A | 0.291637 | -0.0422224 | 0.00904421 | 3.03E-06 | 278804 | 7.81649E-05 | 21.79438186 |
| 48 | rs755726 | T | C | 0.36592 | -0.0423745 | 0.00852878 | 6.75E-07 | 278804 | 8.85315E-05 | 24.68512608 |
| 49 | rs9974484 | C | T | 0.605003 | 0.0410266 | 0.0084202 | 1.10E-06 | 278804 | 8.51433E-05 | 23.74030204 |

Note: SNP, single nucleotide polymorphism; eaf, effect allele frequency; R^2^ = (beta^2^) / (se^2^*N + beta^2^); F = beta^2^ / se^2^

| **Table S6. Genetic instruments for Thoracic spine Pain** | | | | | | | | | | |
| --- | --- | --- | --- | --- | --- | --- | --- | --- | --- | --- |
|  | SNP | other_allele | effect_allele | eaf | beta | se | pval | N | R^2^ | F |
| 1 | rs149532410 | G | A | 0.0041162 | -0.964173 | 0.20649 | 3.02E-06 | 252821 | 8.62306E-05 | 21.80278051 |
| 2 | rs111524558 | C | T | 0.0449266 | 0.216208 | 0.0461069 | 2.74E-06 | 252821 | 8.69683E-05 | 21.98931355 |
| 3 | rs33999424 | A | G | 0.432247 | 0.0920046 | 0.0200112 | 4.27E-06 | 252821 | 8.36033E-05 | 21.13843438 |
| 4 | rs116363196 | T | C | 0.0788182 | 0.17085 | 0.0352677 | 1.27E-06 | 252821 | 9.28159E-05 | 23.46797898 |
| 5 | rs148462270 | T | C | 0.000564323 | 1.46086 | 0.317957 | 4.34E-06 | 252821 | 8.34893E-05 | 21.10961994 |
| 6 | rs1878658 | A | G | 0.135437 | 0.130347 | 0.0282088 | 3.82E-06 | 252821 | 8.44468E-05 | 21.35171957 |
| 7 | rs147363683 | A | G | 0.0526056 | 0.193746 | 0.0418326 | 3.63E-06 | 252821 | 8.48371E-05 | 21.45041872 |
| 8 | rs149457174 | A | G | 0.00124188 | 1.00854 | 0.220581 | 4.83E-06 | 252821 | 8.26801E-05 | 20.90499426 |
| 9 | rs185559827 | G | A | 0.0723012 | -0.192528 | 0.0396327 | 1.19E-06 | 252821 | 9.33312E-05 | 23.59828701 |
| 10 | rs12415721 | T | C | 0.64578 | 0.0975347 | 0.0209406 | 3.20E-06 | 252821 | 8.58005E-05 | 21.69402132 |
| 11 | rs4428983 | A | G | 0.691747 | -0.0977909 | 0.0213023 | 4.42E-06 | 252821 | 8.33479E-05 | 21.07385042 |
| 12 | rs147029050 | G | A | 0.00761854 | 0.497949 | 0.0995258 | 5.64E-07 | 252821 | 9.90016E-05 | 25.0321628 |
| 13 | rs75272226 | G | A | 0.0197729 | 0.306775 | 0.0665267 | 4.00E-06 | 252821 | 8.41004E-05 | 21.26414706 |
| 14 | rs7321408 | T | C | 0.44683 | -0.101867 | 0.0200507 | 3.77E-07 | 252821 | 0.000102082 | 25.81118564 |
| 15 | rs2484981 | T | C | 0.456507 | 0.0928002 | 0.0201766 | 4.24E-06 | 252821 | 8.36666E-05 | 21.15445572 |
| 16 | rs143418431 | G | C | 0.0134873 | 0.368563 | 0.0795746 | 3.63E-06 | 252821 | 8.48447E-05 | 21.45233351 |
| 17 | rs12909263 | T | C | 0.717472 | 0.109962 | 0.0223364 | 8.52E-07 | 252821 | 9.58527E-05 | 24.23588615 |

Note: SNP, single nucleotide polymorphism; eaf, effect allele frequency; R^2^ = (beta^2^) / (se^2^*N + beta^2^); F = beta^2^ / se^2^

| Table S7. Genetic instruments for Low back pain | | | | | | | | | | |
| --- | --- | --- | --- | --- | --- | --- | --- | --- | --- | --- |
|  | SNP | other_allele | effect_allele | eaf | beta | se | pval | N | R^2^ | F |
| 1 | rs6724567 | A | G | 0.55396 | -0.0475544 | 0.00833934 | 1.18E-08 | 342499 | 9.49331E-05 | 32.51756759 |
| 2 | rs7617480 | A | C | 0.838422 | -0.066516 | 0.0112897 | 3.82E-09 | 342499 | 0.000101341 | 34.71262415 |
| 3 | rs6916321 | A | G | 0.708976 | 0.0554918 | 0.00919181 | 1.57E-09 | 342499 | 0.000106402 | 36.44647638 |
| 4 | rs2074483 | G | A | 0.299995 | 0.0526298 | 0.0090467 | 5.97E-09 | 342499 | 9.88054E-05 | 33.84410718 |
| 5 | rs35989721 | T | C | 0.102007 | 0.106318 | 0.0134248 | 2.38E-15 | 342499 | 0.000183088 | 62.71882995 |
| 6 | rs3936186 | G | A | 0.605175 | -0.049576 | 0.00850202 | 5.51E-09 | 342499 | 9.9265E-05 | 34.00155051 |
| 7 | rs4148933 | T | C | 0.555952 | 0.0481292 | 0.00838267 | 9.38E-09 | 342499 | 9.62391E-05 | 32.9649595 |
| 8 | rs11834194 | G | T | 0.157785 | 0.0702323 | 0.0113705 | 6.55E-10 | 342499 | 0.00011138 | 38.15176701 |
| 9 | rs3102180 | A | G | 0.410015 | 0.0471664 | 0.00846994 | 2.57E-08 | 342499 | 9.05328E-05 | 31.01021604 |
| 10 | rs4462678 | A | G | 0.575594 | 0.0631991 | 0.00843681 | 6.84E-14 | 342499 | 0.000163808 | 56.11322761 |

Note: SNP, single nucleotide polymorphism; eaf, effect allele frequency; R^2^ = (beta^2^) / (se^2^*N + beta^2^); F = beta^2^ / se^2^

| **Table S8: Genetic predisposition to Frailty and risk of pain: the results from Mendelian randomization analysis.** | | | | | | | | | | | | | | |
| --- | --- | --- | --- | --- | --- | --- | --- | --- | --- | --- | --- | --- | --- | --- |
| **Exposure** | **Outcome** | **nsnp** | **method** | **OR** | **OR_lci95** | **OR_uci95** | **p** | **Q** | **Q_pval** | **Intercept** | **p_intercept** | **Global test p** | **Correct p** | |
| Frailty phenotype | Pain | 27 | MR Egger | 1.63 | 0.56 | 4.75 | 3.75E-01 |  |  | 0.001 | 0.919 | 4.00E-04 | | NA |
|  |  |  | Weighted median | 1.89 | 1.48 | 2.42 | 3.41E-07 |  |  |  |  |  | |  |
|  |  |  | IVW | 1.73 | 1.37 | 2.17 | 3.54E-06 | 59.96 | 1.70E-04 |  |  |  | |  |
| Frailty phenotype | Join Pain | 28 | MR Egger | 0.83 | 0.18 | 3.92 | 8.15E-01 |  |  | 0.015 | 0.124 | 0.28 | | NA |
|  |  |  | Weighted median | 2.77 | 1.83 | 4.2 | 1.66E-06 |  |  |  |  |  | |  |
|  |  |  | IVW | 2.85 | 2.06 | 3.94 | 2.83E-10 | 32.35 | 2.19E-01 |  |  |  | |  |
| Frailty phenotype | Limb Pain | 29 | MR Egger | 2.84 | 0.3 | 26.67 | 3.70E-01 |  |  | -0.002 | 0.862 | 0.004 | | NA |
|  |  |  | Weighted median | 2.19 | 1.4 | 3.42 | 6.03E-04 |  |  |  |  |  | |  |
|  |  |  | IVW | 2.33 | 1.59 | 3.4 | 1.23E-05 | 54.23 | 2.10E-03 |  |  |  | |  |
| Frailty phenotype | Thoraci spine Pain | 31 | MR Egger | 1.11 | 0.02 | 60.6 | 9.58E-01 |  |  | 0.008 | 0.755 | 0.011 | | 0.28 |
|  |  |  | Weighted median | 1.5 | 0.57 | 3.92 | 4.12E-01 |  |  |  |  |  | |  |
|  |  |  | IVW | 2.08 | 0.91 | 4.78 | 8.28E-02 | 51.4 | 8.84E-03 |  |  |  | |  |
| Frailty phenotype | Low back Pain | 27 | MR Egger | 1.54 | 0.34 | 7.06 | 5.82E-01 |  |  | 0.003 | 0.764 | 0.135 | | NA |
|  |  |  | Weighted median | 2.08 | 1.36 | 3.17 | 7.30E-04 |  |  |  |  |  | |  |
|  |  |  | IVW | 1.94 | 1.39 | 2.7 | 8.27E-05 | 35.38 | 1.04E-01 |  |  |  | |  |
| Frailty index | Pain | 13 | MR Egger | 0.61 | 0.37 | 1.02 | 8.58E-02 |  |  | 0.019 | 0.009 | 0.027 | | 3.72E-04 |
|  |  |  | Weighted median | 1.2 | 0.99 | 1.46 | 6.54E-02 |  |  |  |  |  | |  |
|  |  |  | IVW | 1.36 | 1.15 | 1.6 | 2.43E-04 | 24.01 | 2.03E-02 |  |  |  | |  |
| Frailty index | Join Pain | 14 | MR Egger | 0.54 | 0.18 | 1.65 | 3.02E-01 |  |  | 0.028 | 0.053 | 0.045 | | NA |
|  |  |  | Weighted median | 1.36 | 0.96 | 1.92 | 7.91E-02 |  |  |  |  |  | |  |
|  |  |  | IVW | 1.78 | 1.32 | 2.39 | 1.52E-04 | 24.16 | 2.97E-02 |  |  |  | |  |
| Frailty index | Limb Pain | 14 | MR Egger | 0.57 | 0.21 | 1.53 | 2.84E-01 |  |  | 0.022 | 0.089 | 0.087 | | NA |
|  |  |  | Weighted median | 1.12 | 0.82 | 1.53 | 4.64E-01 |  |  |  |  |  | |  |
|  |  |  | IVW | 1.41 | 1.09 | 1.82 | 8.57E-03 | 20.66 | 7.99E-02 |  |  |  | |  |
| Frailty index | Thoraci spine Pain | 15 | MR Egger | 1.58 | 0.15 | 16.75 | 7.12E-01 |  |  | -0.002 | 0.931 | 0.282 | | NA |
|  |  |  | Weighted median | 1.27 | 0.65 | 2.49 | 4.82E-01 |  |  |  |  |  | |  |
|  |  |  | IVW | 1.42 | 0.83 | 2.42 | 1.97E-01 | 17.12 | 2.50E-01 |  |  |  | |  |
| Frailty index | Low back Pain | 15 | MR Egger | 0.46 | 0.13 | 1.66 | 2.57E-01 |  |  | 0.027 | 0.09 | 3.33E-04 | | 0.024 |
|  |  |  | Weighted median | 1.09 | 0.79 | 1.51 | 6.07E-01 |  |  |  |  |  | |  |
|  |  |  | IVW | 1.48 | 1.07 | 2.05 | 1.85E-02 | 36.93 | 7.56E-04 |  |  |  | |  |

Note: IVW, inverse variance weighted; OR,odds ratio; CI, confidence interval; Q_pval, Cochran Q test pvalue; p_intercept, MR-Egger regression pvalue.

| **Table S9: Genetic predisposition to Pain and risk of Frailty: the results from Mendelian randomization analysis** | | | | | | | | | | | | |  |
| --- | --- | --- | --- | --- | --- | --- | --- | --- | --- | --- | --- | --- | --- |
| **Exposure** | **Outcome** | **nsnp** | **method** | **OR** | **OR_lci95** | **OR_uci95** | **p** | **Q** | **Q_pval** | **Intercept** | **p_intercept** | **Global test p** | **Correct p** |
| Pain | Frailty phenotype | 26 | MR Egger | 0.94 | 0.79 | 1.12 | 5.17E-01 |  |  | 0.004 | 0.124 | 0.053 | NA |
|  |  |  | Weighted median | 1.08 | 1.04 | 1.13 | 3.39E-04 |  |  |  |  |  |  |
|  |  |  | IVW | 1.08 | 1.05 | 1.12 | 4.53E-06 | 38.26 | 4.36E-02 |  |  |  |  |
| Join Pain | Frailty phenotype | 5 | MR Egger | 0.87 | 0.74 | 1.03 | 2.01E-01 |  |  | 0.011 | 0.153 | 0.199 | NA |
|  |  |  | Weighted median | 1.02 | 0.98 | 1.06 | 3.15E-01 |  |  |  |  |  |  |
|  |  |  | IVW | 1.02 | 0.98 | 1.06 | 3.37E-01 | 6.83 | 1.45E-01 |  |  |  |  |
| Limb Pain | Frailty phenotype | 37 | MR Egger | 1 | 0.97 | 1.04 | 8.21E-01 |  |  | 0.001 | 0.181 | 0.065 | NA |
|  |  |  | Weighted median | 1.01 | 0.99 | 1.04 | 2.69E-01 |  |  |  |  |  |  |
|  |  |  | IVW | 1.03 | 1.01 | 1.05 | 8.90E-04 | 50.69 | 5.30E-02 |  |  |  |  |
| Thoraci spine Pain | Frailty phenotype | 9 | MR Egger | 0.98 | 0.92 | 1.04 | 5.27E-01 |  |  | 0.004 | 0.334 | 0.925 | NA |
|  |  |  | Weighted median | 1.01 | 1 | 1.03 | 1.33E-01 |  |  |  |  |  |  |
|  |  |  | IVW | 1.01 | 1 | 1.02 | 9.59E-02 | 3.2 | 9.21E-01 |  |  |  |  |
| Low back Pain | Frailty phenotype | 9 | MR Egger | 1.1 | 0.93 | 1.3 | 2.90E-01 |  |  | -0.003 | 0.524 | 0.119 | NA |
|  |  |  | Weighted median | 1.05 | 1.01 | 1.08 | 7.64E-03 |  |  |  |  |  |  |
|  |  |  | IVW | 1.04 | 1.01 | 1.07 | 1.01E-02 | 13.3 | 1.02E-01 |  |  |  |  |
| Pain | Frailty index | 28 | MR Egger | 0.92 | 0.68 | 1.23 | 5.62E-01 |  |  | 0.007 | 0.159 | 0.068 | NA |
|  |  |  | Weighted median | 1.11 | 1.03 | 1.19 | 6.08E-03 |  |  |  |  |  |  |
|  |  |  | IVW | 1.13 | 1.07 | 1.2 | 2.19E-05 | 38.86 | 6.53E-02 |  |  |  |  |
| Join Pain | Frailty index | 5 | MR Egger | 0.71 | 0.47 | 1.06 | 1.89E-01 |  |  | 0.027 | 0.147 | 0.036 | 0.944 |
|  |  |  | Weighted median | 0.99 | 0.92 | 1.07 | 8.55E-01 |  |  |  |  |  |  |
|  |  |  | IVW | 1.05 | 0.95 | 1.16 | 3.78E-01 | 13.15 | 1.06E-02 |  |  |  |  |
| Limb Pain | Frailty index | 40 | MR Egger | 1.04 | 0.95 | 1.13 | 4.21E-01 |  |  | 0.001 | 0.709 | 3.33E-04 | NA |
|  |  |  | Weighted median | 1.06 | 1.02 | 1.11 | 4.86E-03 |  |  |  |  |  |  |
|  |  |  | IVW | 1.05 | 1.01 | 1.09 | 6.50E-03 | 80.51 | 1.04E-04 |  |  |  |  |
| Thoraci spine Pain | Frailty index | 9 | MR Egger | 0.98 | 0.93 | 1.04 | 5.43E-01 |  |  | 0.004 | 0.259 | 0.359 | NA |
|  |  |  | Weighted median | 1.01 | 0.98 | 1.04 | 5.49E-01 |  |  |  |  |  |  |
|  |  |  | IVW | 1.01 | 0.99 | 1.04 | 2.85E-01 | 9.06 | 3.37E-01 |  |  |  |  |
| Low back Pain | Frailty index | 8 | MR Egger | 1.1 | 0.88 | 1.39 | 4.32E-01 |  |  | 0.001 | 0.974 | 0.643 | NA |
|  |  |  | Weighted median | 1.12 | 1.05 | 1.19 | 5.91E-04 |  |  |  |  |  |  |
|  |  |  | IVW | 1.11 | 1.06 | 1.16 | 1.24E-05 | 5.5 | 5.99E-01 |  |  |  |  |

Note: IVW, inverse variance weighted; OR,odds ratio; CI, confidence interval; Q_pval, Cochran Q test pvalue; p_intercept, MR-Egger regression pvalue.
