# Supplementary material for: The vicious cycle of frailty and pain: a two-sided causal relationship revealed
Source: Front Med (Lausanne). 2024 Sep 9;11:1396328. doi: 10.3389/fmed.2024.1396328 (PMC11416971; doi:10.3389/fmed.2024.1396328)
Supplement: Supplementary file 2 [file Data_Sheet_1.pdf]

Content

Figure S1 Scatterplot of Mendelian randomization (MR) estimates of genetic risk of Frailty phenotype on Pain(A) ,Join Pain (B), Limb Pain(C),Thoraci spine Pain(D) and Low back Pain(E).

Figure S2 Scatterplot of MR estimates of genetic risk of Frailty index on Pain(A) ,Join Pain (B), Limb Pain(C),Thoraci spine Pain(D) and Low back Pain(E).

Figure S3 Scatterplot of MR estimates of genetic risk of Pain on Frailty phenotype(A) , Frailty index(B).

Figure S4 Scatterplot of MR estimates of genetic risk of Join Pain on Frailty phenotype(A) , Frailty index(B).

Figure S5 Scatterplot of MR estimates of genetic risk of Limb Pain on Frailty phenotype(A) , Frailty index(B).

Figure S6 Scatterplot ofMR estimates of genetic risk of Thoraci spine Pain on Frailty phenotype(A) , Frailty index(B).

Figure S7 Scatterplot of MR estimates of genetic risk of Low back Pain on Frailty phenotype(A) , Frailty index(B).

Figure S8 Funnal plot to assess heterogeneity for Frailty phenotype and Pain(A) ,Join Pain (B), Limb Pain(C),Thoraci spine Pain(D), Low back Pain(E).

Figure S9 Funnal plot to assess heterogeneity for Frailty index and Pain(A) ,Join Pain (B), Limb Pain(C),Thoraci spine Pain(D), Low back Pain(E).

Figure S10 Funnal plot to assess heterogeneity for Pain and Frailty phenotype and Pain(A),Frailty index(B).

Figure S11 Funnal plot to assess heterogeneity for Join Pain and Frailty phenotype and Pain(A),Frailty index(B).

Figure S12 Funnal plot to assess heterogeneity for Limb Pain and Frailty phenotype and Pain(A),Frailty index(B).

Figure S13 Funnal plot to assess heterogeneity for Thoraci spine Pain and Frailty phenotype and Pain(A),Frailty index(B).

Figure S14 Funnal plot to assess heterogeneity for Low back Pain and Frailty phenotype and Pain(A),Frailty index(B).

Figure S15 MR leave-one-out sensitivity analysis for Frailty phenotype and Pain(A) ,Join Pain (B), Limb Pain(C),Thoraci spine Pain(D), Low back Pain(E).

Figure S16 MR leave-one-out sensitivity analysis for Frailty index and Pain(A) ,Join Pain (B), Limb Pain(C),Thoraci spine Pain(D), Low back Pain(E).

Figure S17 MR leave-one-out sensitivity analysis for Pain and Frailty phenotype(A) ,Frailty index (B).

Figure S18 MR leave-one-out sensitivity analysis for Join Pain (B) and Frailty phenotype(A) ,Frailty index (B).

Figure S19 MR leave-one-out sensitivity analysis for Limb Pain and Frailty phenotype(A) ,Frailty index (B).
Figure S20 MR leave-one-out sensitivity analysis for Thoraci spine Pain and Frailty phenotype(A) ,Frailty index (B).
Figure S21 MR leave-one-out sensitivity analysis for Low back Pain and Frailty phenotype(A) ,Frailty index (B).

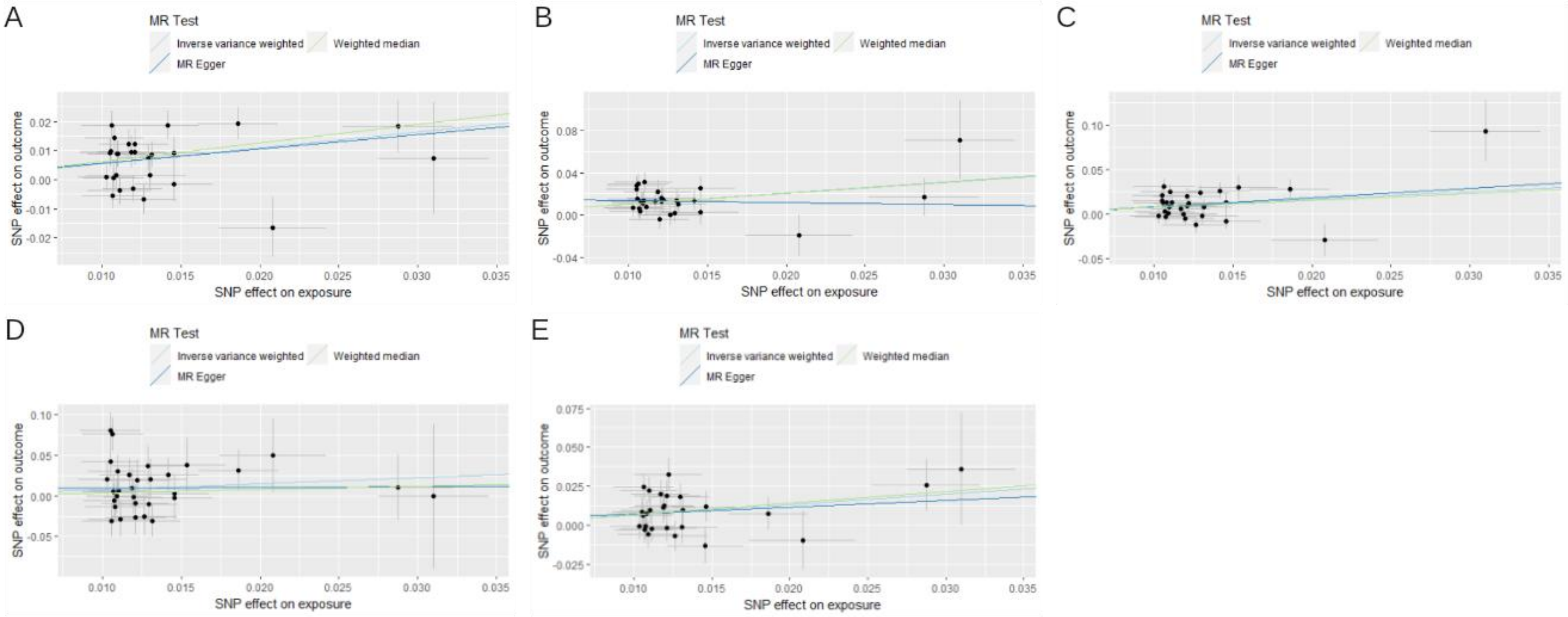

Figure S1：Scatterplot of Mendelian randomization (MR) estimates of genetic risk of Frailty phenotype on Pain(A) ,Join Pain (B), Limb Pain(C),Thoraci spine Pain(D) and Low back Pain(E).

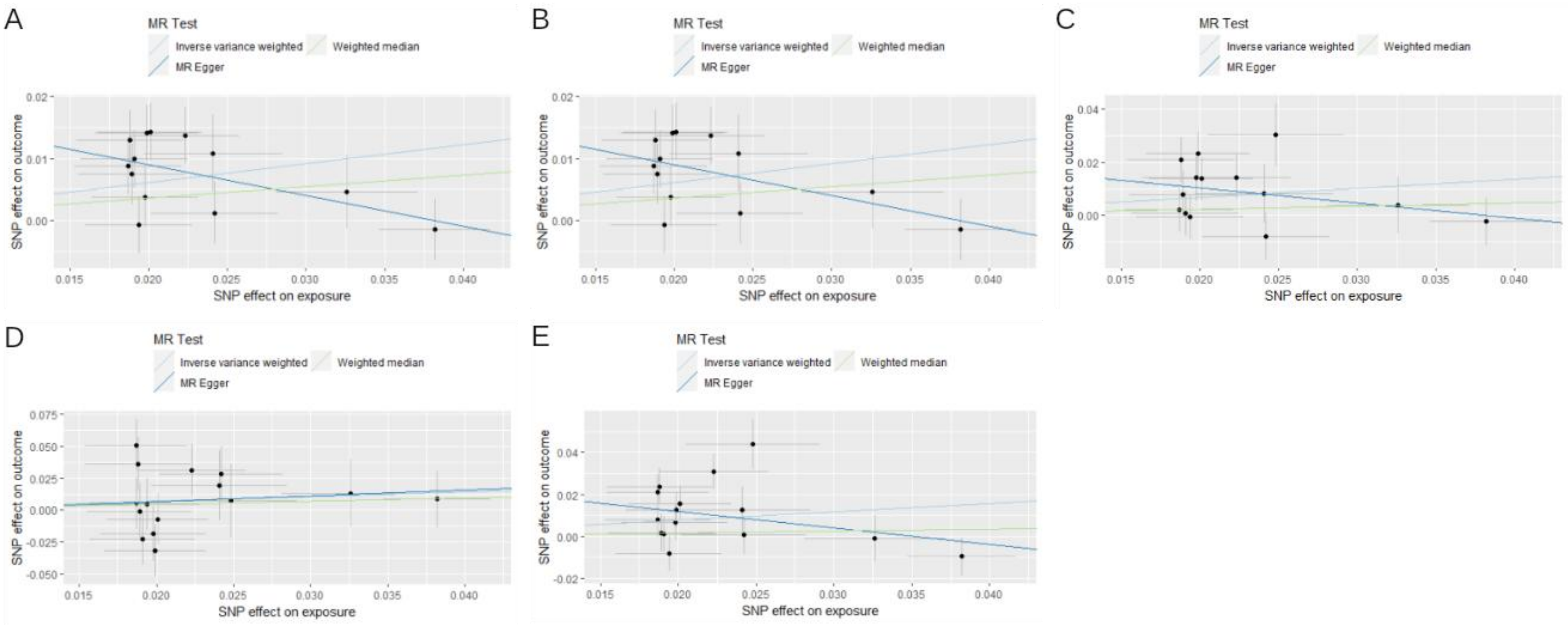

Figure S2：Scatterplot of Mendelian randomization (MR) estimates of genetic risk of Frailty index on Pain(A) ,Join Pain (B), Limb Pain(C),Thoraci spine Pain(D) and Low back Pain(E).

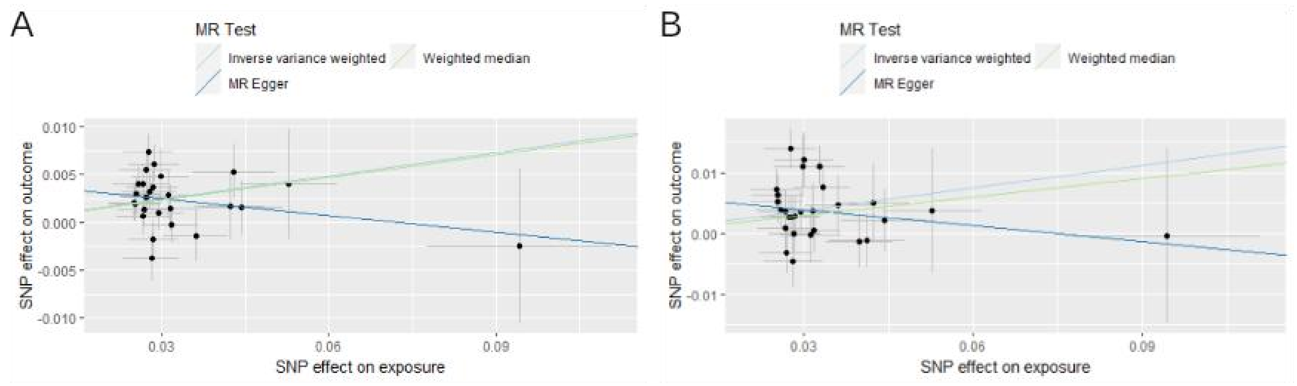

Figure S3：Scatterplot of Mendelian randomization (MR) estimates of genetic risk of Pain on Frailty phenotype(A) , Frailty index(B).

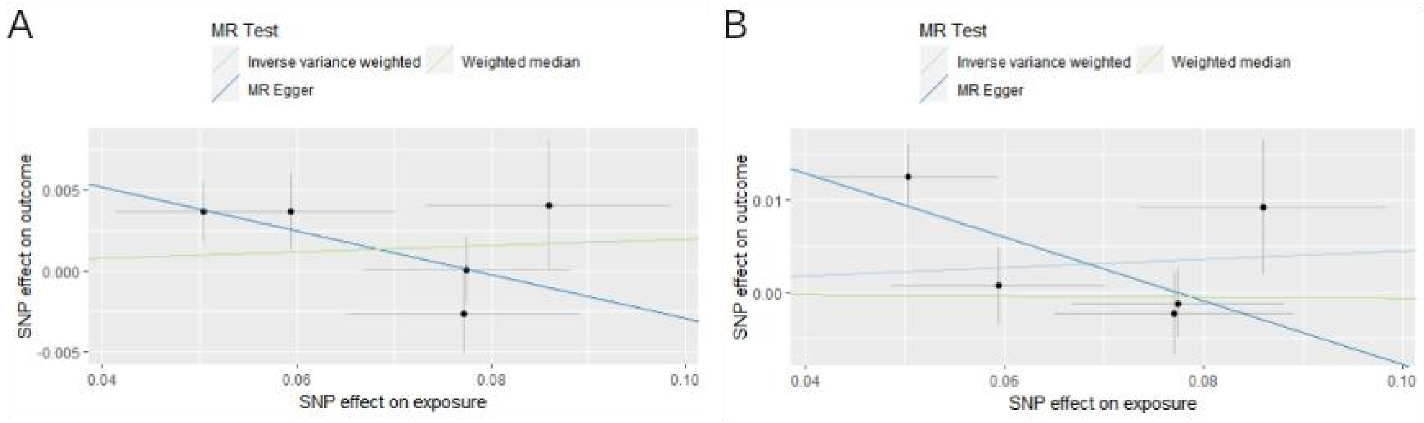

Figure S4：Scatterplot of Mendelian randomization (MR) estimates of genetic risk of Join Pain on Frailty phenotype(A) , Frailty index(B).

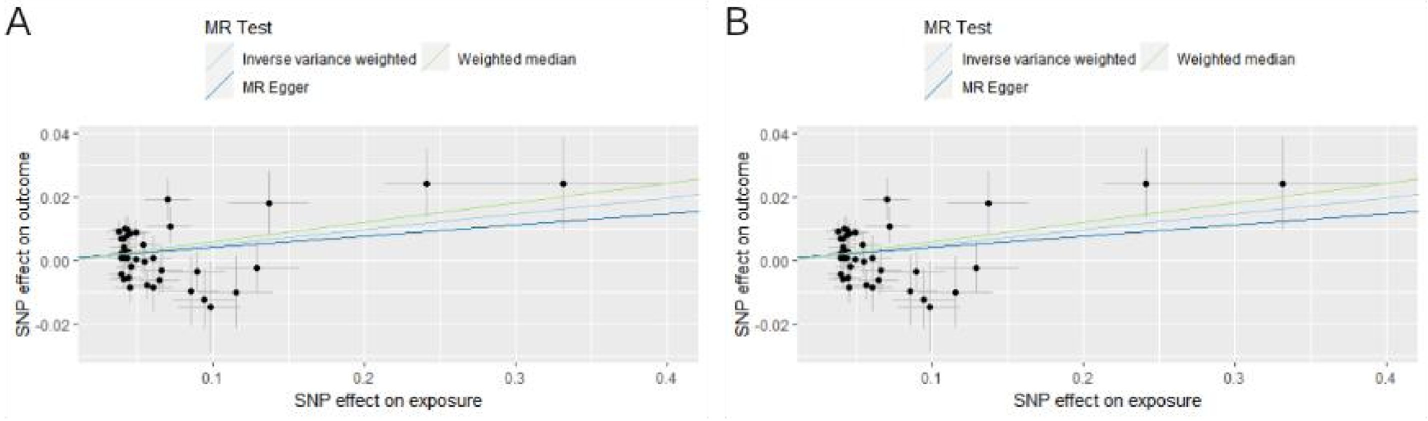

Figure S5： Scatterplot of Mendelian randomization (MR) estimates of genetic risk of Limb Pain on Frailty phenotype(A) , Frailty index(B).

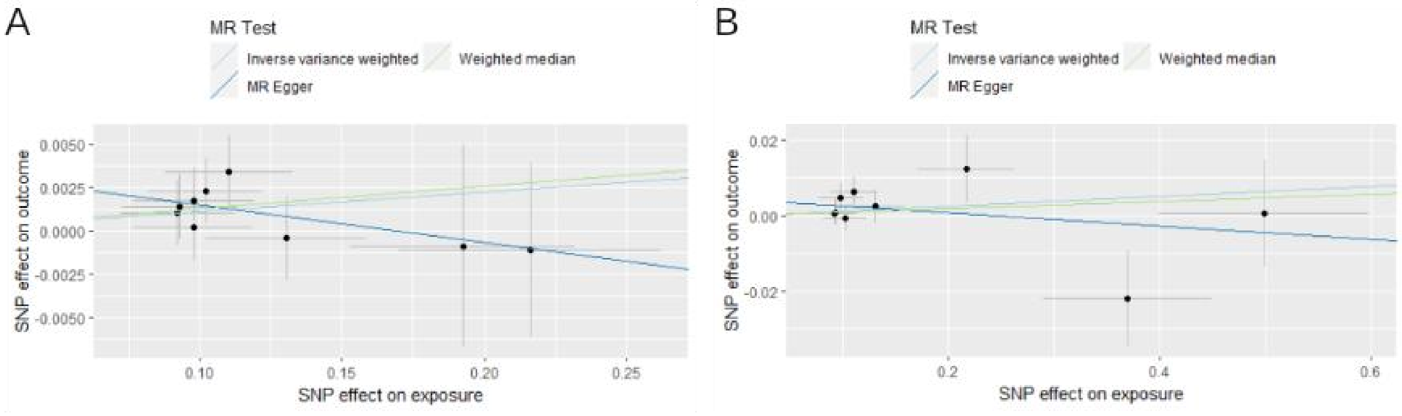

Figure S6： Scatterplot of Mendelian randomization (MR) estimates of genetic risk of Thoraci spine Pain on Frailty phenotype(A) , Frailty index(B).

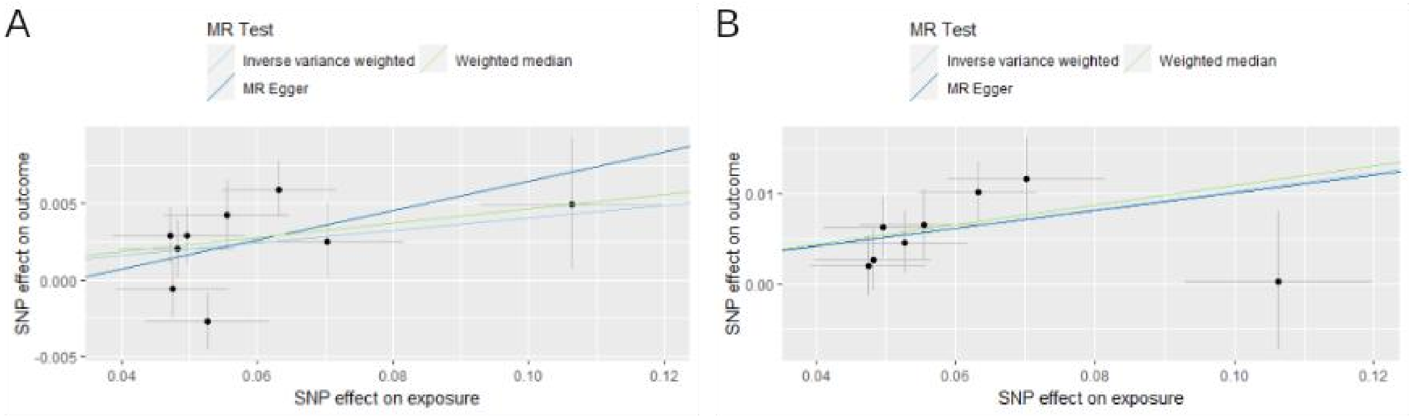

Figure S7：Scatterplot of Mendelian randomization (MR) estimates of genetic risk of Low back Pain on Frailty phenotype(A) , Frailty index(B).

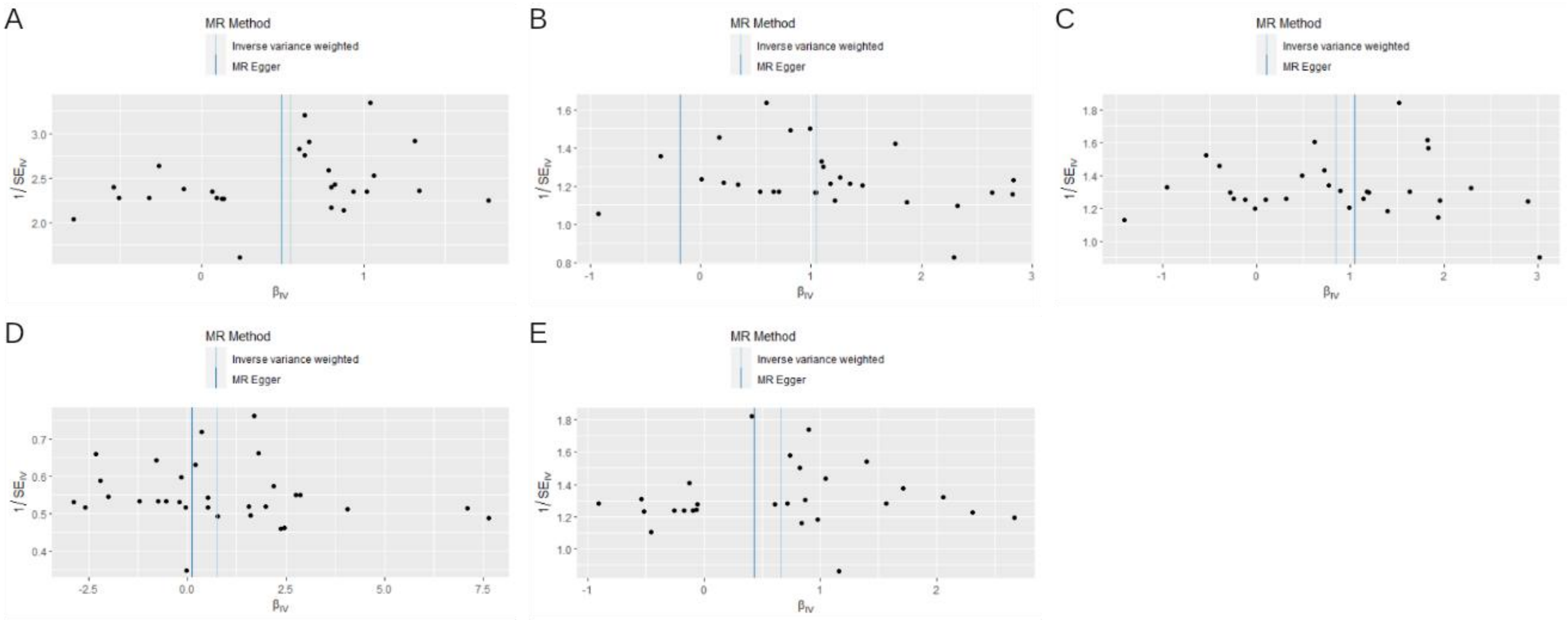

Figure S8 Funnal plot to assess heterogeneity for Frailty phenotype and Pain(A) ,Join Pain (B), Limb Pain(C),Thoraci spine Pain(D), Low back Pain(E).

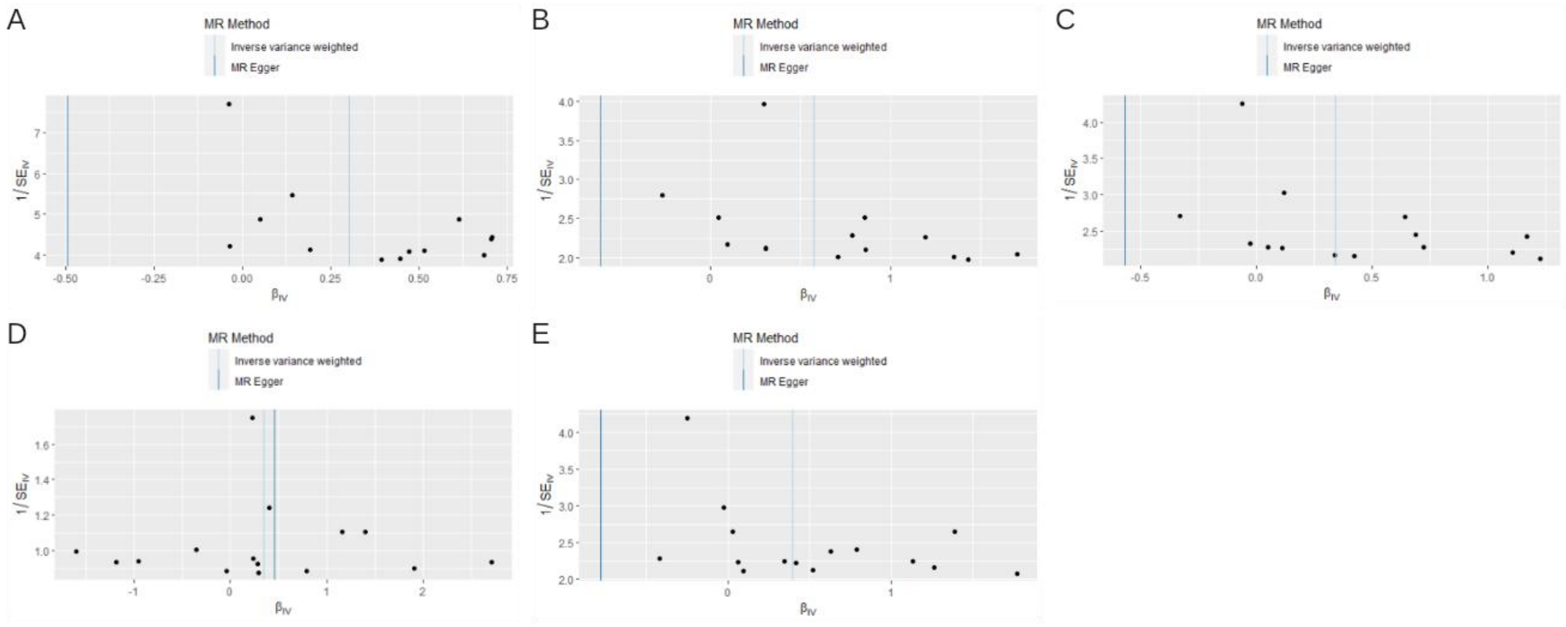

Figure S9 Funnal plot to assess heterogeneity for Frailty index and Pain(A) ,Join Pain (B), Limb Pain(C),Thoraci spine Pain(D), Low back Pain(E).

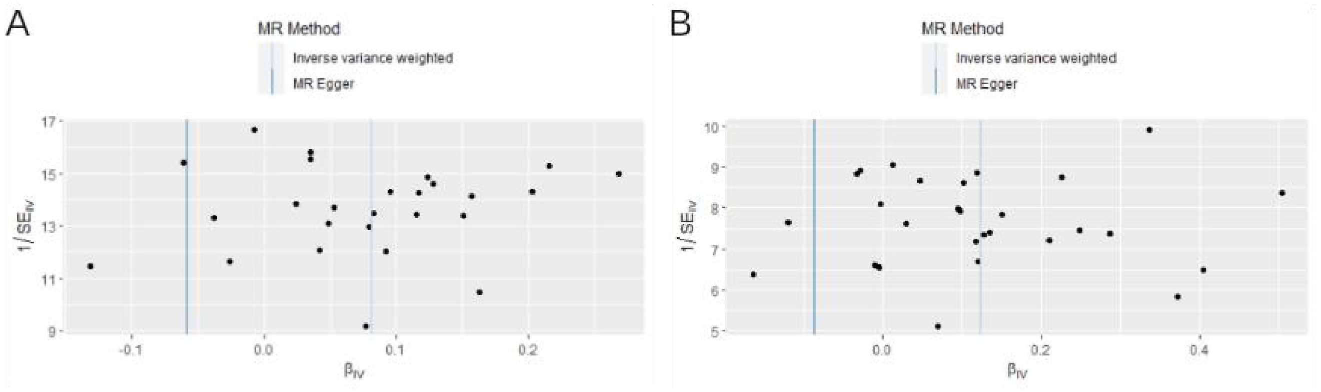

Figure S10 Funnal plot to assess heterogeneity for Pain and Frailty phenotype and Pain(A),Frailty index(B).

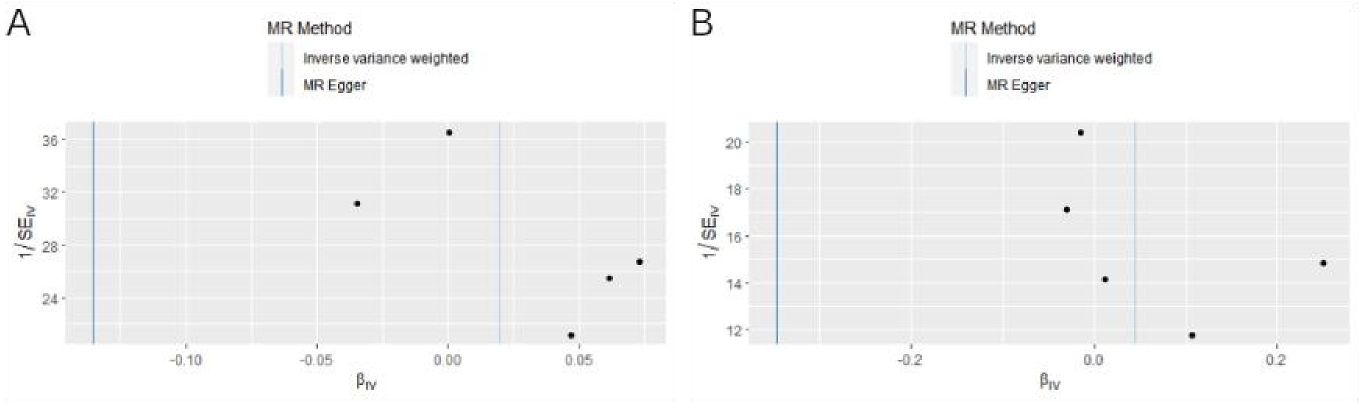

Figure S11 Funnal plot to assess heterogeneity for Join Pain and Frailty phenotype and Pain(A),Frailty index(B).

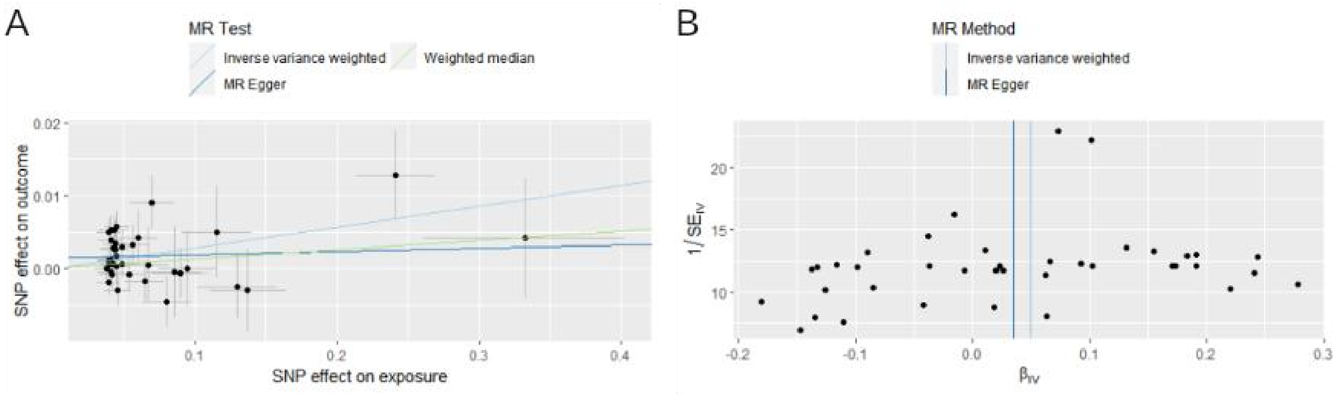

Figure S12   Funnal plot to assess heterogeneity for Limb Pain and Frailty phenotype and Pain(A),Frailty index(B).

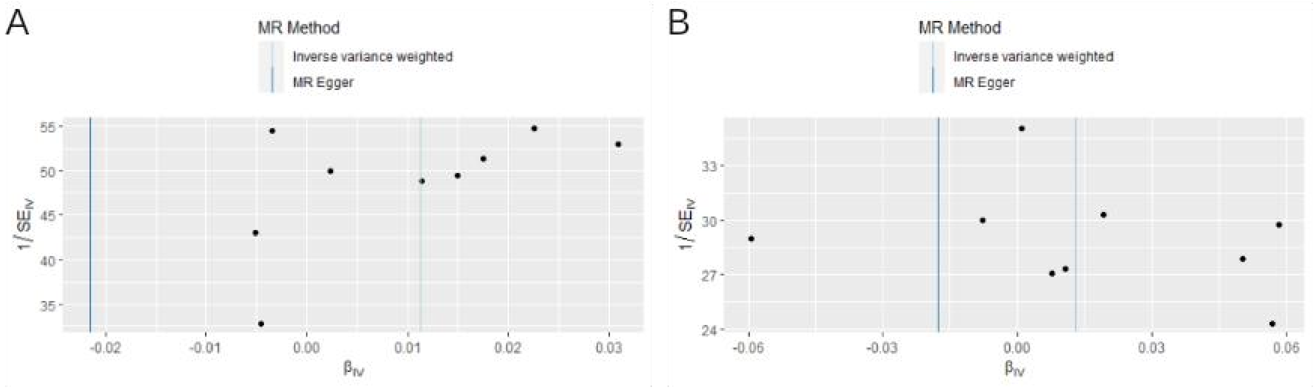

Figure S13   Funnal plot to assess heterogeneity for Thoraci spine Pain and Frailty phenotype and Pain(A),Frailty index(B).

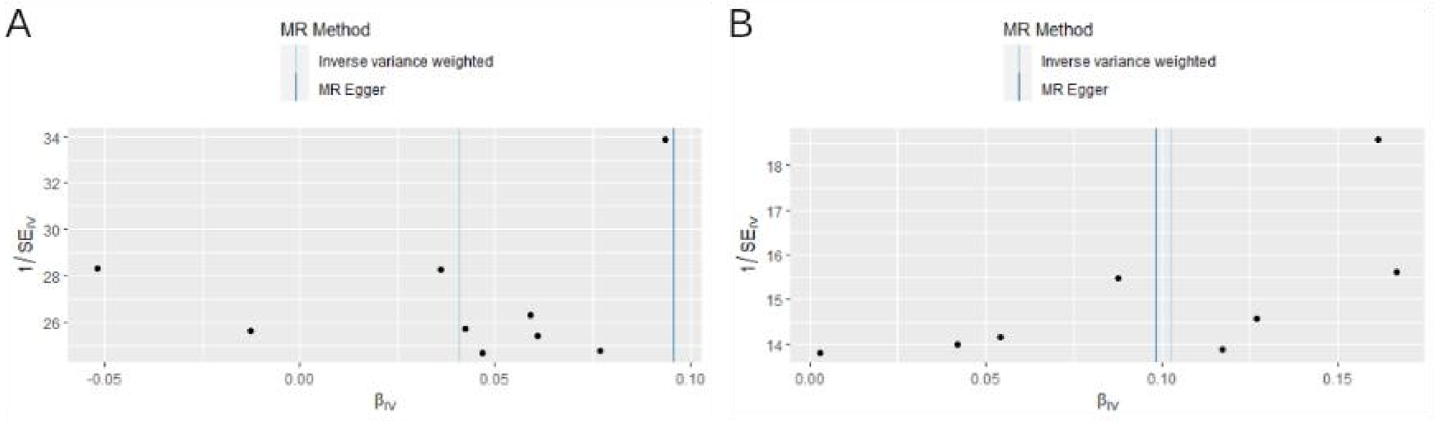

Figure S14 Funnal plot to assess heterogeneity for Low back Pain and Frailty phenotype and Pain(A),Frailty index(B).

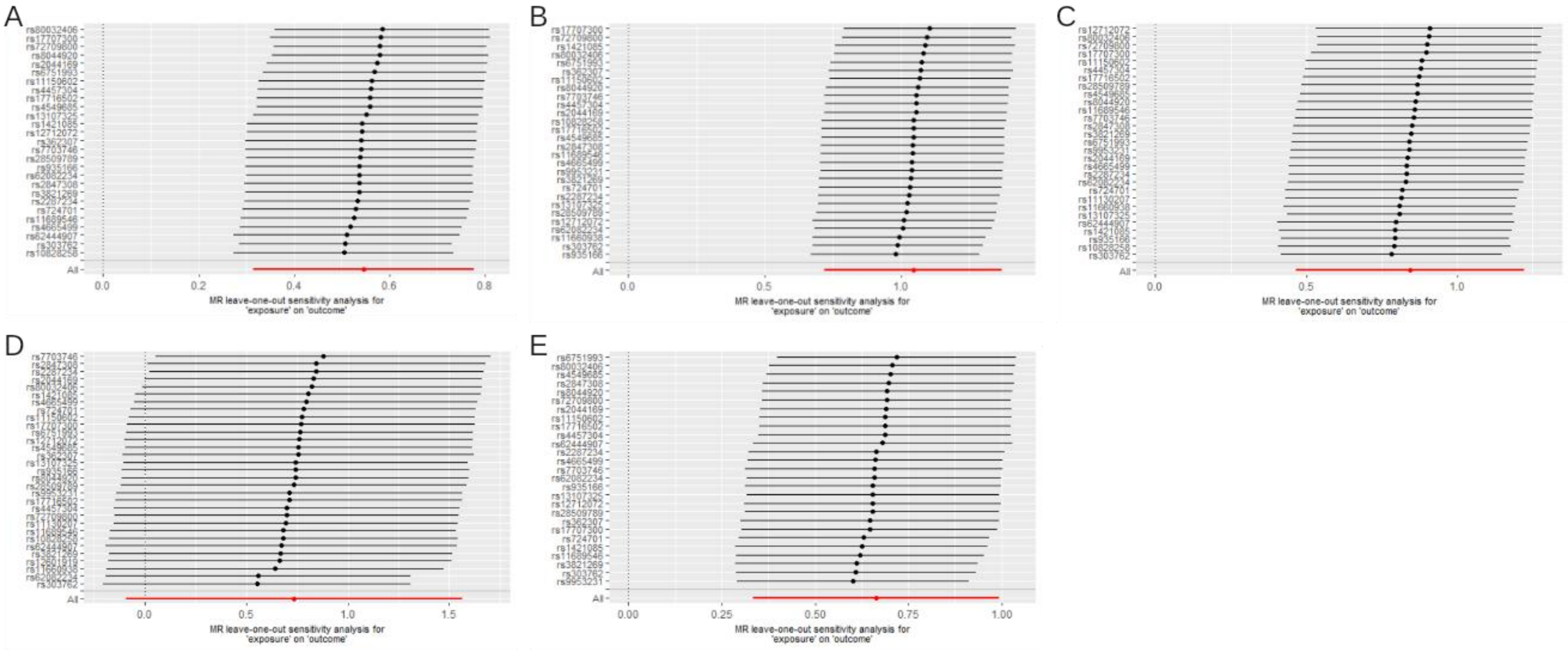

Figure S15 MR leave-one-out sensitivity analysis for Frailty phenotype and Pain(A) ,Join Pain (B), Limb Pain(C),Thoraci spine Pain(D), Low back Pain(E).

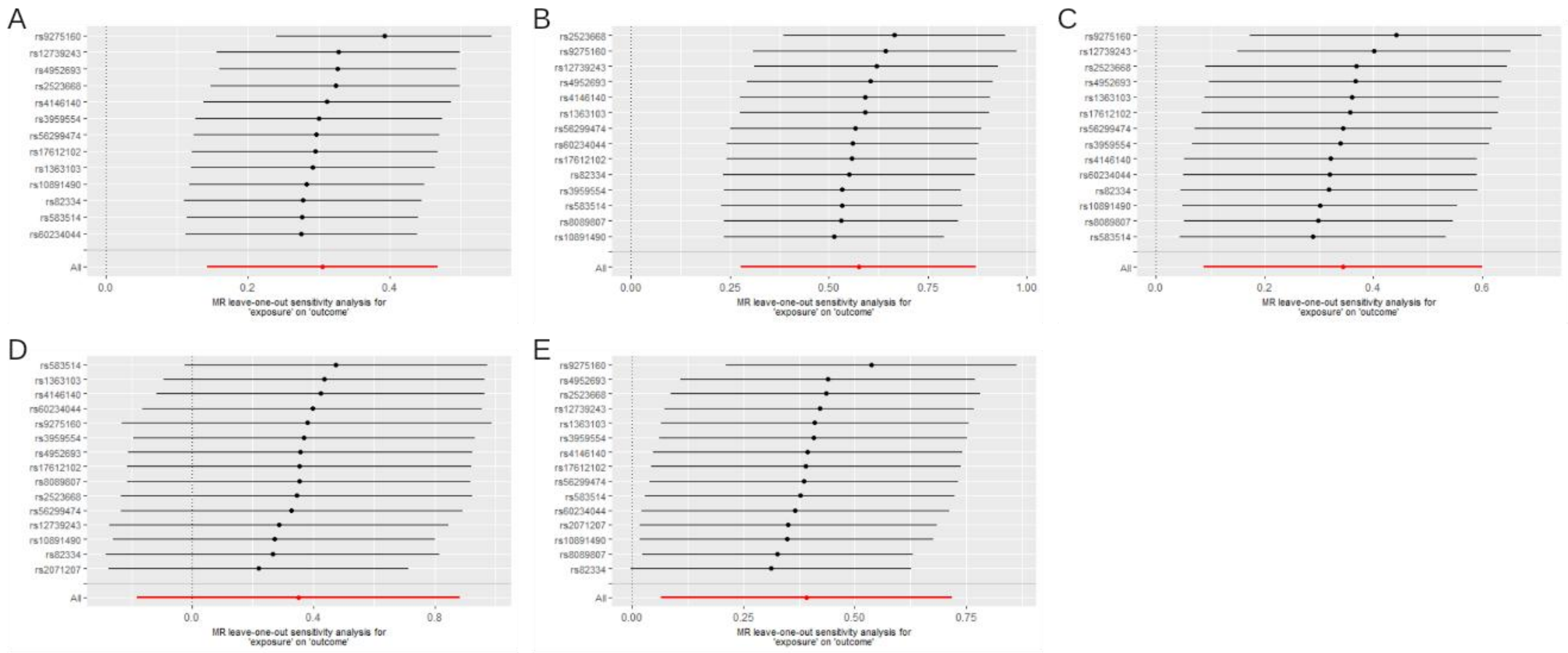

Figure S16 MR leave-one-out sensitivity analysis for Frailty index and Pain(A) ,Join Pain (B), Limb Pain(C),Thoraci spine Pain(D), Low back Pain(E).

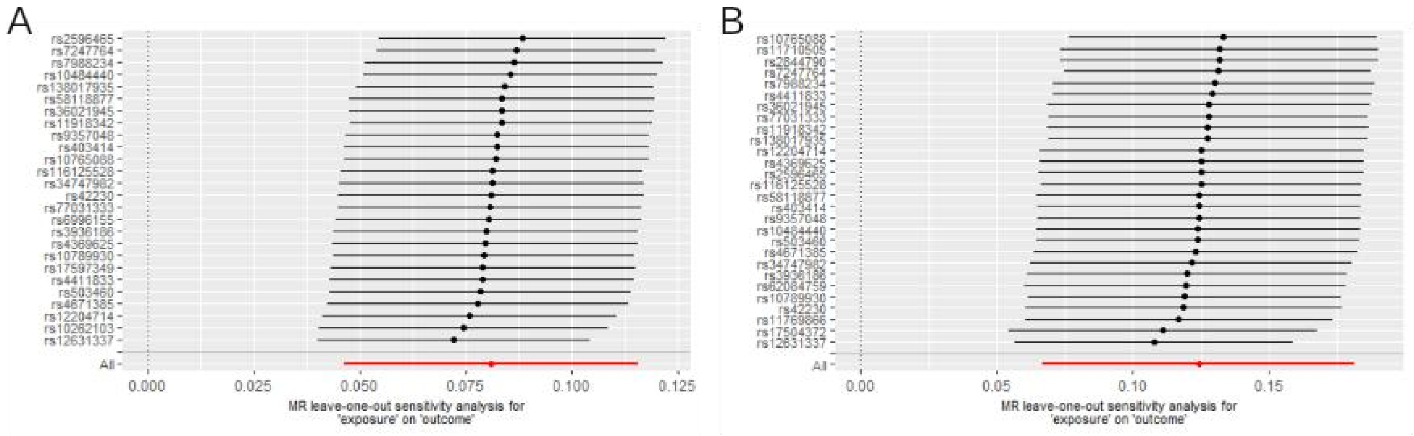

Figure S17 MR leave-one-out sensitivity analysis for Pain and Frailty phenotype(A) ,Frailty index (B).

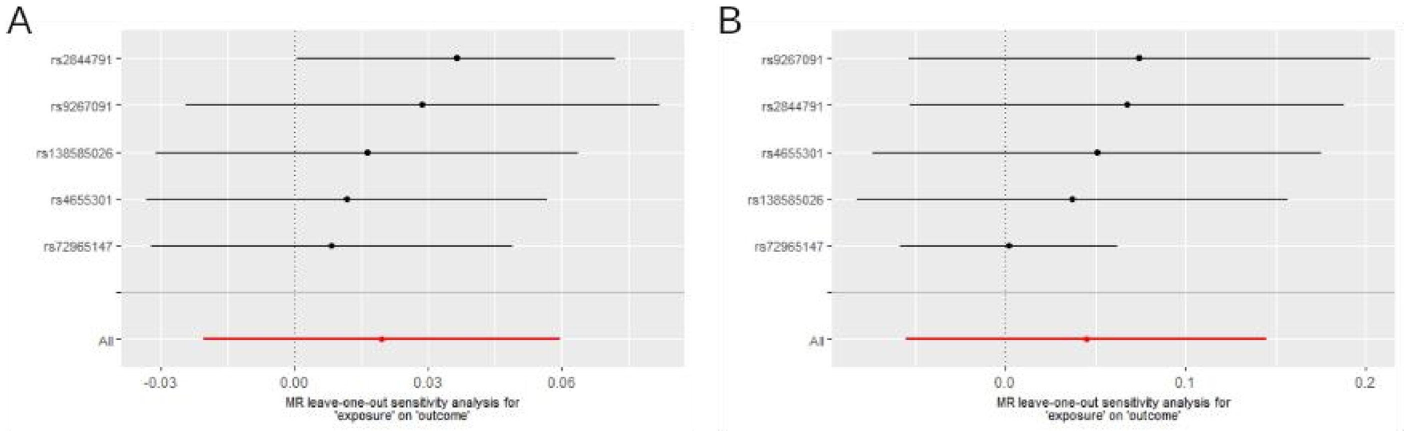

Figure S18 MR leave-one-out sensitivity analysis for Join Pain and Frailty phenotype(A) ,Frailty index (B).

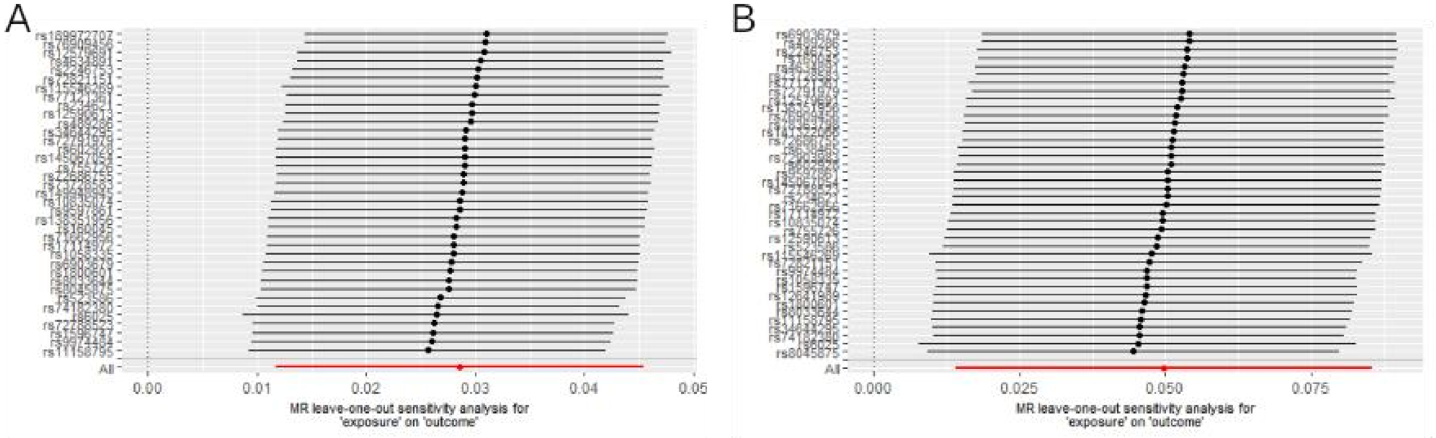

Figure S19 MR leave-one-out sensitivity analysis for Limb Pain and Frailty phenotype(A) ,Frailty index (B).

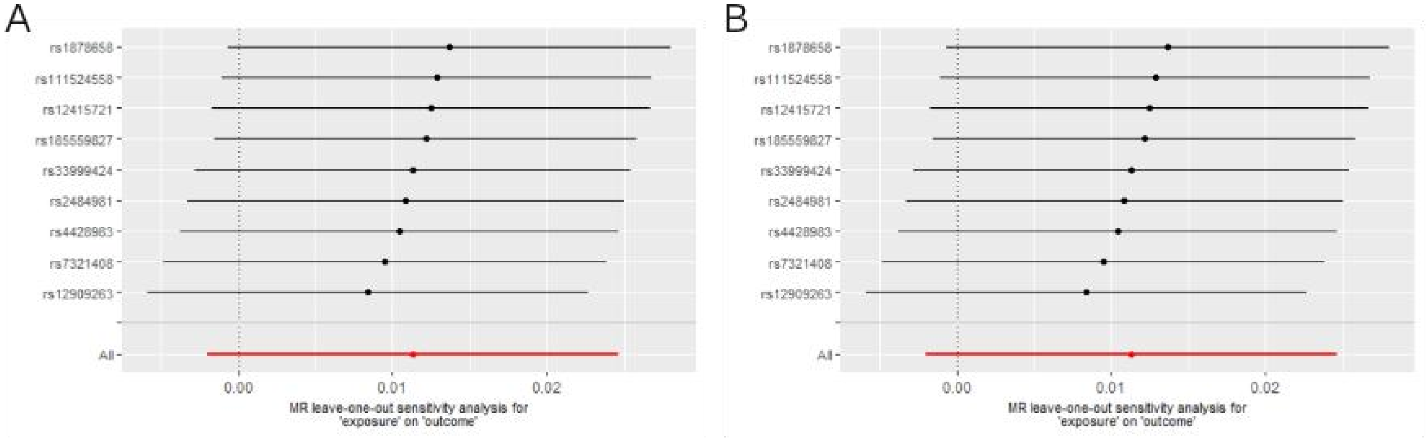

Figure S20 MR leave-one-out sensitivity analysis for Thoraci spine Pain and Frailty phenotype(A) ,Frailty index (B).

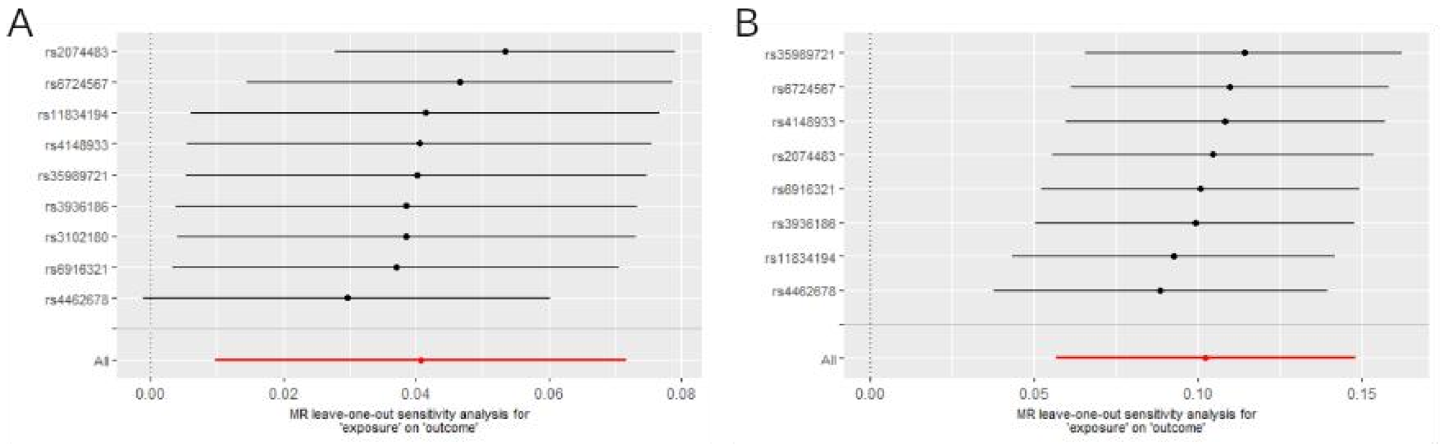

Figure S21 MR leave-one-out sensitivity analysis for Low back Pain and Frailty phenotype(A) ,Frailty index (B).
